# *XACT* Noncoding RNA Competes with *XIST* in the Control of X Chromosome Activity during Human Early Development

**DOI:** 10.1016/j.stem.2016.10.014

**Published:** 2017-01-05

**Authors:** Céline Vallot, Catherine Patrat, Amanda J. Collier, Christophe Huret, Miguel Casanova, Tharvesh M. Liyakat Ali, Matteo Tosolini, Nelly Frydman, Edith Heard, Peter J. Rugg-Gunn, Claire Rougeulle

**Affiliations:** 1 Sorbonne Paris Cité, Epigenetics and Cell Fate, UMR 7216 Centre National de la Recherche Scientifique (CNRS), Université Paris Diderot, 75013 Paris, France; 2 Mammalian Developmental Epigenetics Group, Institut Curie, PSL Research University, CNRS UMR3215, INSERM U934, 75005 Paris, France; 3 Université Paris Diderot, Sorbonne Paris Cité, 75018 Paris, France; 4 Reproductive Biology Department, AP-HP, Bichat-Claude Bernard Hospital, 75018 Paris, France; 5 Epigenetics Programme, The Babraham Institute, Cambridge CB22 3AT, UK; 6 Wellcome Trust – Medical Research Council Cambridge Stem Cell Institute, University of Cambridge, Cambridge CB2 1QR, UK; 7 Centre for Trophoblast Research, University of Cambridge, Cambridge CB2 3EG, UK; 8 Université Paris-Sud, Clamart 92140, France; 9 Unit of Reproductive Biology, AP-HP, Hôpital Antoine Béclère, Clamart 92141, France

**Keywords:** human X chromosome inactivation, long noncoding RNA, naive pluripotency, preimplantation development, dosage compensation, XIST, XACT

## Abstract

Sex chromosome dosage compensation is essential in most metazoans, but the developmental timing and underlying mechanisms vary significantly, even among placental mammals. Here we identify human-specific mechanisms regulating X chromosome activity in early embryonic development. Single-cell RNA sequencing and imaging revealed co-activation and accumulation of the long noncoding RNAs (lncRNAs) *XACT* and *XIST* on active X chromosomes in both early human pre-implantation embryos and naive human embryonic stem cells. In these contexts, the *XIST* RNA adopts an unusual, highly dispersed organization, which may explain why it does not trigger X chromosome inactivation at this stage. Functional studies in transgenic mouse cells show that *XACT* influences *XIST* accumulation in *cis*. Our findings therefore suggest a mechanism involving antagonistic activity of *XIST* and *XACT* in controlling X chromosome activity in early human embryos, and they highlight the contribution of rapidly evolving lncRNAs to species-specific developmental mechanisms.

## Highlights

• *XACT* and *XIST* simultaneously coat active X chromosomes in human embryos
• Naive hESCs capture the pre-inactive state with *XIST* and *XACT* co-accumulation
• *XIST* adopts a dispersed configuration in pre-inactive cells in vitro and in vivo
• *XACT* prevents *XIST* accumulation in *cis* in a mouse heterologous system

## Graphical Abstract

**Figure:**
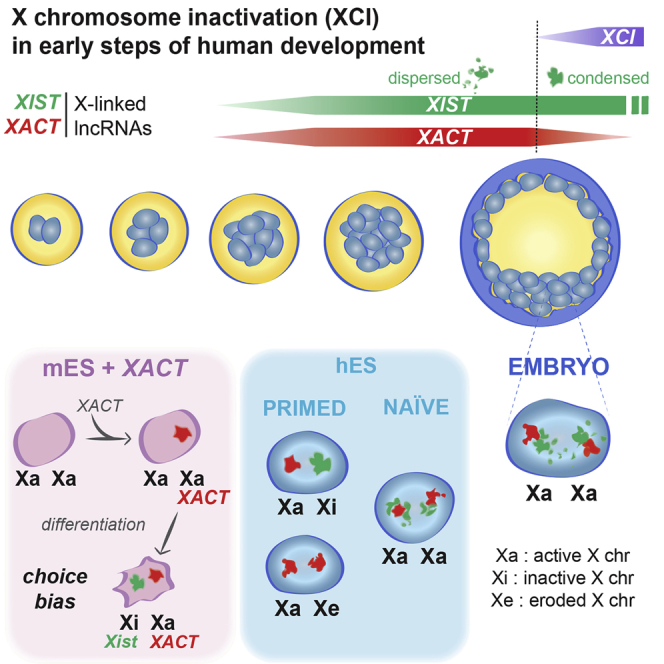

## Teaser

Rougeulle and colleagues show that, in human pre-implantation embryos and naive human embryonic stem cells, the lncRNAs *XIST* and *XACT* accumulate together on active X chromosomes. Functional data suggest that *XACT* restrains *XIST* activity before the initiation of X inactivation takes place.

## Introduction

In mammals, the activity of the X chromosomes has to be tightly controlled to accommodate the disequilibrium of X-linked gene dosage between males and females. This is achieved through compensatory mechanisms, which serve to equalize X chromosome expression between sexes and relative to autosomes (Disteche, 2016). While X chromosome dosage compensation is essential for proper development, the underlying strategies vary extensively between species. In the mouse, for instance, X chromosome inactivation (XCI) is initiated rapidly after zygotic genome activation (ZGA), several divisions prior to blastocyst implantation. In contrast, human pre-implantation development proceeds in the apparent absence of XCI; instead, dosage compensation is transiently achieved by reducing the expression of both X chromosomes in females (Petropoulos et al., 2016). The mechanisms underlying these differences are still poorly understood.

XCI is triggered by the accumulation of the long noncoding RNA (lncRNA) *XIST* on the chromosome, which recruits protein complexes involved in chromatin remodeling, chromosome structuration, and nuclear organization (Chu et al., 2015, McHugh et al., 2015, Minajigi et al., 2015). Strikingly, however, in human pre-implantation embryos, *XIST* accumulates on every X chromosome in both males and females without inducing robust transcriptional repression or enrichment of H3K27me3, a hallmark of the inactive state (Okamoto et al., 2011). This unusual configuration suggests the existence, in embryos, of mechanisms preventing *XIST*-mediated XCI, which would to some extent be specific to the human, since in the mouse *Xist* accumulation systematically results in the inactivation of the coated chromosome.

We recently have identified *XACT*, an X-linked lncRNA that coats active X chromosomes in human pluripotent stem cells. *XACT* is weakly conserved across mammals and is absent from the mouse, suggesting that it could fulfill a primate-specific function (Vallot et al., 2013). Insight into such function came from the analysis of XCI status and its instability in human embryonic stem cells (hESCs). XCI has indeed been established in most hESCs derived so far, but this status is unstable and hallmarks of the inactive state are spontaneously and irreversibly lost in culture. This erosion of XCI is characterized by the loss of *XIST* expression, loss of H3K27me3 enrichment, DNA hypomethylation, and partial gene reactivation (Mekhoubad et al., 2012, Nazor et al., 2012, Vallot et al., 2015). Accumulation of *XACT* is also a feature of the eroded X and occurs early during the erosion process, prior to the loss of *XIST* and to gene reactivation (Vallot et al., 2015). This order of events suggests that *XACT* could participate in the instability of XCI in hESCs by influencing *XIST* expression, *XIST* RNA activity, or localization.

Here we show that *XACT* is expressed in human pre-implantation embryos, where it accumulates, together with *XIST*, on active X chromosomes. We also report an in vitro context of naive pluripotency that recapitulates the in vivo situation, thus defining a unique pre-inactivation state in human development. Functional evidences further indicate that *XACT* influences *XIST* accumulation in *cis*, suggesting that dosage compensation establishment in human involves the antagonistic action of two lncRNAs.

## Results

### *XIST* and *XACT* Expression Profiling from Single-Cell RNA-Seq Human Pre-implantation Embryo Datasets

**Figure 1:**
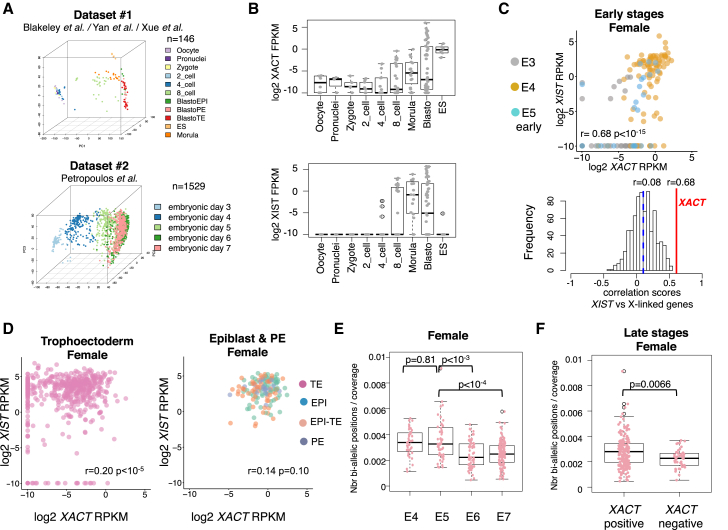
*XACT* and *XIST* Are Co-expressed in the Early Stages of Human Development (A) Principal component analysis illustrates the developmental trajectory within the two datasets used in this study, based on the n = 1,000 most variant GENCODE genes within each dataset. (B) Boxplot of *XACT* and *XIST* expression levels (log2 FPKM) according to developmental stage, from dataset 1. (C) Upper panel: plot of *XACT* versus *XIST* expression levels (log2 reads per kilobase per million mapped reads [RPKM]) in early-stage female cells of dataset 2 (E3, E4, and early E5), with corresponding Spearman correlation score and p value. Lower panel: distribution of Spearman correlation scores between *XIST* and each X-linked gene expression level. The median correlation score is indicated as a blue dashed line and a red line indicates the correlation score with *XACT* expression levels. (D) Plots of *XACT* versus *XIST* expression levels (log2 RPKM) in late-stage female cells of dataset 2 (E5, E6, and E7), in trophectoderm (left panel) and epiblast and primitive endoderm cells, as assessed by a 75-gene signature shown in Figure S1C. The corresponding Spearman correlation score and p value are indicated. (E) Boxplot of the number of X-linked bi-allelic positions normalized by coverage according to day of development for dataset 2. The p values of a Wilcoxon rank test comparing distributions between stages are indicated above the boxplot. (F) Boxplot of the number of X-linked bi-allelic positions normalized by coverage for *XACT*-positive and -negative cells in female late-stage blastocysts. The p value of a Wilcoxon rank test comparing both distributions is indicated above the boxplot.

To investigate the biological relevance of *XACT* and its putative function in XCI, we probed its expression in vivo, in the early stages of human development. We analyzed four independent sets of single-cell RNA sequencing (RNA-seq) data generated from human pre-implantation embryos, which we grouped into two datasets (Figure 1A) based on the methods used to classify embryos. In dataset 1 (Blakeley et al., 2015, Xue et al., 2013, Yan et al., 2013), embryos (41 embryos; n = 146 cells) are classified by developmental stages, ranging from oocyte to blastocyst, whereas in dataset 2 (Petropoulos et al., 2016), embryos (88 embryos; n = 1,529 cells) are classified according to the day of development, ranging from embryonic day E3 to E7.

The major burst of zygotic *XIST* and *XACT* expression occurred concomitantly between the four-cell and the eight-cell stages (Figure 1B). *XIST* was undetectable prior to these stages, while there was a modest maternal contribution for *XACT* (average fragments per kilobase per million mapped reads [FPKM] = 0.005 from oocyte to two-cell stage). The expression of *XIST* and *XACT* was strongly correlated at early stages of development (from E3 to early E5) in both female and male embryos, suggesting that the two noncoding genes, which are located 40 Mb apart on the X, might be co-regulated (Figure 1C; Figure S1B). This correlation was likely significant and not only due to ZGA, as it was not observed for any other X-linked genes (lower panel, Figure 1C). However, *XIST*-*XACT* correlation did not persist in later-stage embryos (from E5 to E7, Figure S1D). In females, *XIST* and *XACT* were maintained co-expressed in epiblast (EPI and EPI-TE) and primitive endoderm (PE) cells. In contrast, trophectoderm (TE) cells displayed variable *XACT* levels (Figure 1D) while maintaining stable *XIST* expression. Such a pattern suggests that, once initiated, the expression of *XIST* and *XACT* can be regulated independently, in agreement with the persistence of *XIST* expression from the inactive X chromosome in differentiated cells where *XACT* is repressed (Vallot et al., 2013).

We next exploited both datasets to follow the dynamics of *XACT* and *XIST* expression in the context of XCI. Applying a dedicated pipeline based on Genome Analysis Toolkit (GATK) tools (McKenna et al., 2010), we used the normalized number of X-linked bi-allelic positions as a marker of X chromosome activity in female cells (Figures S1E and S1F). The number of bi-allelic positions on the X decreased significantly with developmental stage in both datasets compared to several autosomes (Figure 1E; Figures S1G and S1H), indicative of a potential initiation of X chromosome silencing. Although this contrasts somewhat with a recent report that concluded an absence of XCI at these stages (Petropoulos et al., 2016), the discrepancy may be due to the choice of threshold for calling bi-allelic positions (i.e., allelic ratio, Figure S1I). Altogether, these findings suggest the existence of an expression imbalance between the two X chromosomes starting at E6, which can be interpreted as an initiation of XCI. Strikingly, *XACT*-negative cells were the ones displaying the lowest numbers of X-linked bi-allelic positions (Figure 1F, p = 0.0066), potentially linking in time the repression of *XACT* to the initiation of XCI.

### *XIST* and *XACT* Co-accumulate on X Chromosomes in Pre-implantation Embryos

**Figure 2:**
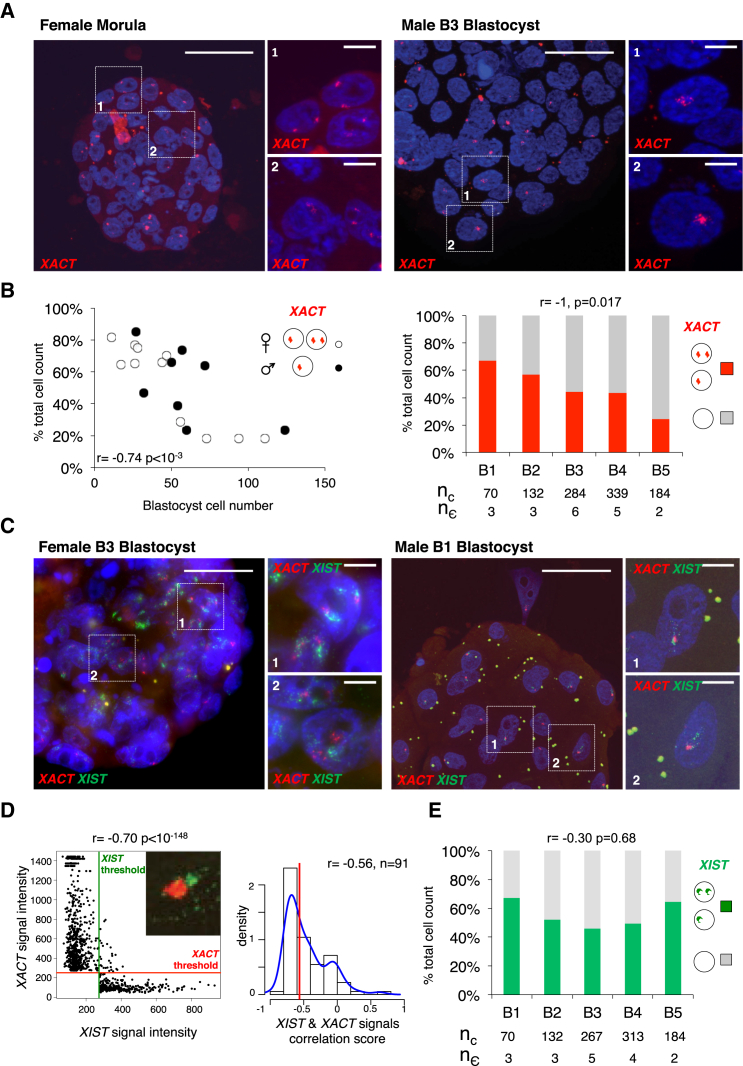
*XACT* Co-accumulates with *XIST* on the X Chromosome(s) in Human Pre-implantation Embryos (A) Examples of female and male embryos analyzed by RNA-FISH to detect *XACT* expression. (B) Left panel: the proportion of cells per male (dark circles) and female (white circles) blastocyst displaying *XACT* expression was plotted against blastocyst cell number. Right panel: the percentage of cells with *XACT* expression at each blastocyst stage (right), from B1 to B5, is shown. Below the graphs are indicated total cell number (n_c_) and the number of embryos (n_ε_) analyzed. The Spearman correlation score between the proportion of *XACT*-positive cells and the developmental stage is indicated. (C) Simultaneous analysis of *XIST* (in green) and *XACT* (in red) expression by RNA-FISH in female and male blastocysts. White scale bars represent 50 μm for total embryos and 5 μm for zooms. (D) Left panel: scatterplot representing the intensity of *XIST* versus *XACT* signal above respective thresholds for one nucleus. The red horizontal line and the green vertical line represent computed thresholds for *XACT*- and *XIST*-associated images, respectively. Signals were compared using a Spearman correlation test. Right panel: distribution of Spearman correlation scores for n = 91 nuclei is shown. A red line indicates the median correlation score and a blue line displays the density plot. (E) Percentage of cells with *XIST* accumulation at each blastocyst stage, from B1 to B5. Below the graphs are indicated total cell number (n_c_) and the number of embryos (n_ε_) analyzed. The Spearman correlation score between the proportion of *XIST*-positive cells and the developmental stage is indicated.

Single-cell RNA-seq analysis thus revealed that *XACT* and *XIST* can be simultaneously expressed within a given cell and likely from a given chromosome, as extrapolated from male samples. The known ability of these two noncoding transcripts to individually coat the chromosome from which they are expressed prompted us to investigate their nuclear distributions at the single-cell level in human pre-implantation embryos. This also enabled us to refine our understanding of the timing and allelism of expression and the localization of *XACT* and *XIST* by precise developmental scoring of embryos, through the use of a blastocyst morphological classification (Gardner et al., 2000) and the assessment of total cell counts for each embryo. RNA-fluorescence in situ hybridization (FISH) analysis of one female morula and eight male and 11 female blastocysts not only confirmed the expression of *XACT* at these developmental stages but also further demonstrated that *XACT* retains its ability to form an RNA cloud in vivo (Figure 2A). *XACT* coated the single X chromosome in males and one or both Xs in the majority of cells of female morula and early blastocysts, with a tendency to shift from bi- to mono-allelic expression as female blastocysts increased in cell number (Figure S2A). The overall proportion of cells with *XACT* expression decreased as development progressed (Figure 2B). Our prior RNA-seq analysis together with visual observation of labeled blastocysts suggests that *XACT*-negative cells are from the TE.

Simultaneous RNA-FISH for *XIST* and *XACT* in male and female blastocysts showed that both transcripts were often co-expressed from the X chromosome and accumulated in large domains within the nucleus (Figure 2C). *XIST* and *XACT* RNAs were, however, differentially distributed and the two signals barely overlapped (Figure 2C, zooms, Figure 2D), indicating that *XIST* and *XACT* transcripts occupy distinct nuclear territories. While the expression of *XIST* remained constant from the early (B1) to late/hatching (B5) blastocyst stage (Figure 2E), the proportion of cells with co-accumulation of *XACT* and *XIST* decreased according to the blastocyst cell number (Figure S2B), in agreement with the loss of *XACT* expression in a subset of blastocyst cells. In addition, no recurrent pattern could be identified when the relative allelic expression of *XIST* and *XACT* were compared (Figure S2C), confirming that, once activated, *XIST* and *XACT* can be regulated independently and suggesting some degree of stochasticity in their expression in blastocysts.

### *XIST* and *XACT* Co-accumulate on Active X Chromosome in Naive Pluripotent Stem Cells

In vivo pre-inactivation status in human is thus characterized by the co-accumulation of *XACT* and *XIST* on active X chromosomes, in both males and females. hESCs in a pre-inactive state have been reported (Gafni et al., 2013, Lengner et al., 2010, Tomoda et al., 2012, Ware et al., 2014), but this status appears to be difficult to generate and maintain in culture. In addition, determining the X chromosome activity status in hESCs could prove challenging, given the spontaneous propensity of the inactive X to undergo partial reactivation in cultured hESCs and the resulting confusion between pre-inactive and eroded states (Vallot et al., 2015). More importantly, none of the naive-state hESCs reported so far have displayed *XIST* expression, as would be predicted from the data presented here and from previous analysis of *XIST* RNA status in human embryos (Okamoto et al., 2011). Additional protocols to capture naive hESCs have been described recently; in these cases, the epigenetic, metabolic, and transcriptomic signatures were strongly indicative of naive-state pluripotency (Takashima et al., 2014, Theunissen et al., 2014), but the status of the X chromosome had not been investigated in depth.

**Figure 3:**
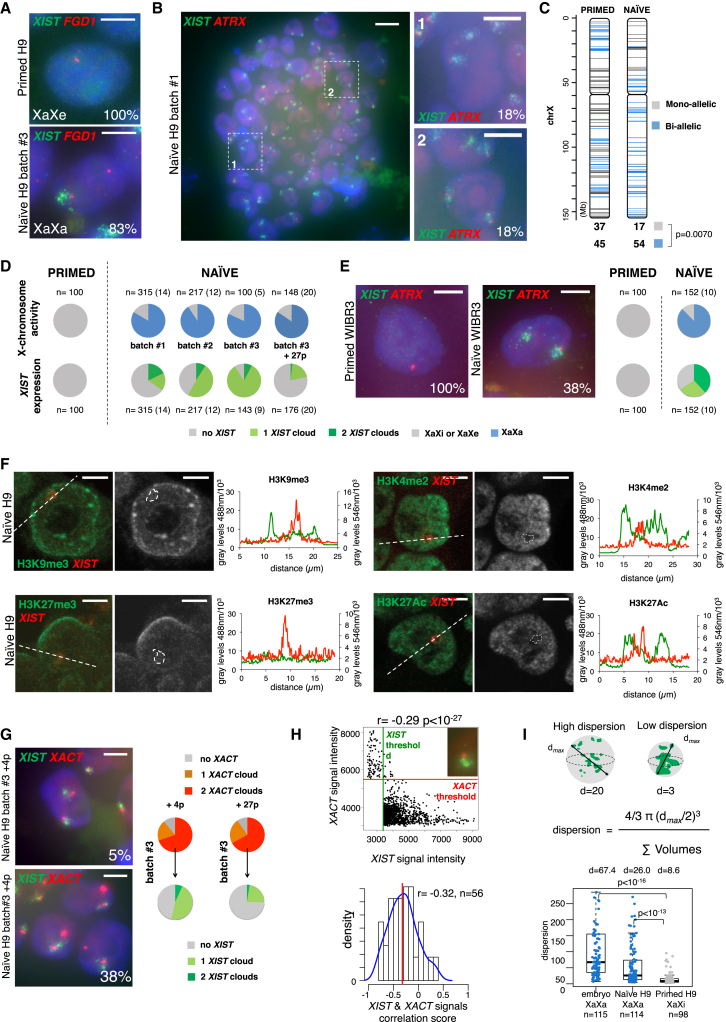
Naive hESCs Carry Active X Chromosomes Coated by *XIST* (A) Analysis of *XIST* (in green) and *FGD1* (in red) expression by RNA-FISH in female H9 primed hESCs and their naive derivatives. The numbers indicate the percentage of cells with the displayed expression pattern. Primed cells have one active X chromosome (Xa) and one X chromosome that has undergone erosion of XCI (Xe). Naive cells carry two active Xs (XaXa). The white scale bars represent 5 μm. (B) Analysis of *XIST* (in green) and *ATRX* (in red) expression by RNA-FISH in naive H9 cells. Panels 1 and 2 are zooms taken within the colony displayed on the left. The white scale bars represent 10 μm for the image of the colony (left panel) and 5 μm for the zooms (right panels). (C) Allelic analysis of RNA-seq data generated from H9 primed (left) and naive (right) cells (Takashima et al., 2014). Informative positions gathered from the H9 genome (Vallot et al., 2015) showing mono-allelic expression are indicated in gray, those with bi-allelic expression in blue. Numbers of mono-allelic and bi-allelic genes in primed and naive cells are indicated beneath each cartoon. Numbers of bi- and mono-allelically expressed genes were compared using a Fisher’s exact test. (D) Upper panel: quantification of XaXa (blue) and XaXi/XaXe (gray) cells in primed and in several batches of naive H9 cells. The XaXa status was defined based on bi-allelic expression of *FGD1*. n, the number of cells counted; in brackets are indicated the number of colonies. Lower panel: similar quantification of cells with zero (gray), one (light green), and two (dark green) *XIST* RNA clouds is shown. p, passage number. (E) Assessment of *ATRX* (in red) and *XIST* (in green) expression in primed and naive female WIBR3 cells. Quantification is as in (D), except that the XaXa status in that case was defined based on bi-allelic expression of *ATRX*. (F) Confocal analysis of immunofluorescence (IF) for H3K9me3, H3K27me3, H3K4me2, and H3K27Ac (in green) coupled to *XIST* RNA-FISH (in red). A representative image is shown for each histone mark, and panels on the right display quantification of gray levels for IF and RNA-FISH signals along the white dashed lines within a single z section. The white scale bar represents 5 μm. (G) Assessment of *XIST* (in green) and *XACT* (in red) expression by RNA-FISH and quantification of the observed profiles in two different batches of naive H9 cells. p, passage number. (H) Left panel: scatterplot representing the intensity of *XIST* versus *XACT* signal above respective thresholds for one nucleus. The red horizontal line and the green vertical line represent computed thresholds for *XACT*- and *XIST*-associated images, respectively. Signals were compared using a Spearman correlation test. Right panel: distribution of Spearman correlation scores for n = 56 nuclei. A red line indicates the median correlation score and a blue line displays the density plot. (I) Left panel: model for the computation of a dispersion value for each *XIST* signal. Right panel: boxplot illustrates the distribution of the dispersion of *XIST* RNA-FISH signal in embryos and in primed and naive H9 and WIBR2 cells. Median dispersions are indicated above the boxplot for each group, Wilcoxon rank test was used to compare distributions of *XIST* signal dispersion among primed H9 cells, naive H9 cells, and embryo cells.

We first studied in primed H9 female hESCs and in their naive derivatives (Takashima et al., 2014) the expression of *ATRX* and *FGD1*, since these two X-linked genes resist XCI erosion (Vallot et al., 2015) yet are expressed from the two Xs in female pre-implantation embryos (Okamoto et al., 2011). Both genes were indeed mono-allelically expressed in the parental, primed hESCs. In striking contrast, two pinpoints for *ATRX* and *FGD1* were detected in 84% and 83%, respectively, of the naive cells, demonstrating that the conversion from primed to naive states was accompanied by substantial X chromosome reactivation (XCR) (Figures 3A and 3B). The presence of two active Xs in naive cells was further confirmed by allelic analysis of RNA-seq datasets (GEO: GSE60945 of Takashima et al., 2014), which revealed an increase in bi-allelically expressed genes in the naive state as compared to primed cells (from 55% to 76%, p = 0.0070, Figure 3C). More strikingly, while *XIST* was fully and uniformly repressed in the parental primed cells, indicating that these cells had undergone XCI erosion, *XIST* was strongly upregulated in naive hESCs, and it accumulated in the nucleus in over 80% of naive H9 on one or two Xs (Figures 3A and 3B). The expression of *XIST* was, however, highly unstable, and the percentage of cells with two or even one *XIST* RNA cloud displayed high batch-to-batch variability. This variation occurred independently of the activity status of the X chromosome, which remained remarkably constant (Figure 3D). Bi-allelic *ATRX* expression and *XIST* accumulation on one or both Xs was similarly observed in an independent naive cell line WIBR3 (Figure 3E) (Theunissen et al., 2014). We also investigated whether the commercially available RSeT-defined medium (STEMCELL Technologies) could efficiently revert XCI, but we did not detect bi-allelic expression of *ATRX* or *XIST* expression, revealing that XCR did not occur in these conditions (Figures S3A–S3D).

We probed the chromatin landscape of the X chromosomes in naive female H9 hESCs. Confocal analyses of immunofluorescence (IF) profiles for various histone marks combined to *XIST* RNA-FISH first showed an absence of H3K27me3 and H3K9me3 within the *XIST* RNA domain (Figure 3F), indicating that *XIST* accumulation in this context was not sufficient to trigger recruitment of repressive histone marks. This was reminiscent of the situation in human embryos where no H3K27me3 enrichment was detected on the *XIST*-coated chromosome (Okamoto et al., 2011). Analysis of published chromatin immunoprecipitation sequencing (ChIP-seq) datasets (Theunissen et al., 2014, Theunissen et al., 2016) confirmed the lack of H3K27me3 and H3K9me3 enrichment on the X in naive hESCs compared to the Xi in *XIST*-expressing primed cells (Figure S3E). Strikingly, IF analysis of H3K4me2 and H3K27Ac further revealed that the X chromosomes in naive hESCs, although active, resided in nuclear territories relatively devoid of active histone marks (Figure 3F).

We next investigated *XACT* expression in naive hESCs by RNA-FISH. *XACT* was expressed and accumulated on every X chromosome in the majority of naive H9 and WIBR3 cells (Figure 3G; Figure S3F), and this pattern was stably maintained in culture (Figure 3G). In cells in which *XIST* also was expressed, the two lncRNAs, although produced from the same chromosome(s), accumulated in distinct nuclear domains; as in embryos, no overlap could be detected between *XIST* and *XACT* signals (Figure 3H, median Spearman correlation score r = −0.32). Reinforcing the similarity between naive hESC and pre-implantation embryos, qualitative analysis revealed a much more dispersed *XIST* RNA-FISH signal in conditions where the Xs were active (embryos and naive hESCs) than in *XIST*-expressing primed hESCs (Wilcoxon rank test p value < 10^−16^ and p < 10^−13^, respectively), in which *XIST* coated an inactive X chromosome (Figure 3I). The different distribution of the *XIST* signal in naive versus primed pluripotent cells might underlie its limited ability to silence the chromosome in *cis*.

### *XACT* Expression Influences *XIST* Accumulation in a Transgenic Context

**Figure 4:**
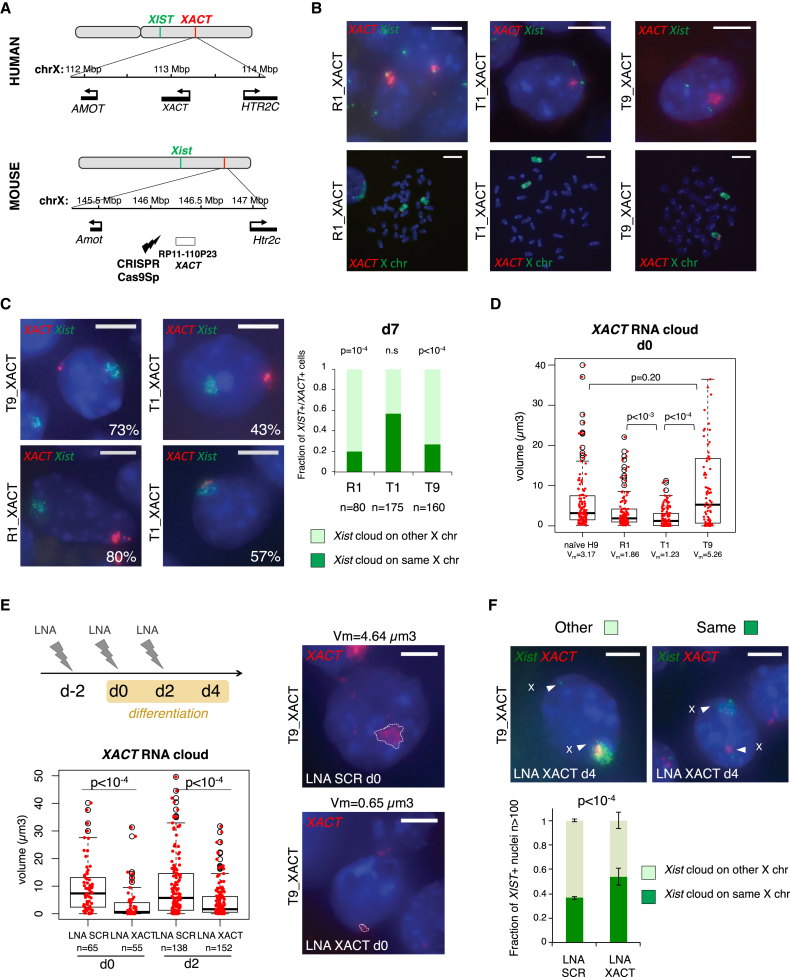
Inserting *XACT* on a Mouse X Chromosome Influences the Choice of the Inactive X (A) Map of the *XACT* locus in human and of the syntenic region on the mouse X chromosome into which the *XACT* BAC was integrated. (B) Assessments of *XACT* and *Xist* expression in interphase nuclei of undifferentiated mouse ESCs (upper panels) and of their localization on metaphase chromosomes (lower panels) in clones with random (R1) or targeted (T1 and T9) integration of *XACT*. (C) Assessment of the chromosome of origin for *Xist* expression related to *XACT* integration in *Xist*- and *XACT*-positive cells at day 7 of differentiation, using RNA-FISH for *Xist* (in green) and *XACT* (in red). Barplot on the right panel shows the quantification of the respective expression patterns in one representative experiment, which were compared using a Fisher’s exact test. (D) Boxplot representing the distribution of the volume of the *XACT* RNA-FISH signal in naive H9 and in transgenic mESC clones. Wilcoxon rank test was used to compare the volume of *XACT* signals. (E) LNA Gapmer-mediated *XACT* KD. Upper panel: timeline indicating the KD strategy during differentiation of the T9 transgenic clone is shown. Three rounds of LNA transfection were performed: one 2 days before the induction of differentiation (d-2), one concomitantly with the launching of differentiation (d0), and the last at day 2 (d2) of differentiation. Lower panel: boxplot representing the distribution of the volume of the *XACT* RNA-FISH signal in cells transfected with either scramble or *XACT* LNA, both at d0 and d2 of differentiation, is shown. Wilcoxon rank test was used to compare the volume of *XACT* signals between KD and control cells. Representative *XACT* RNA-FISH images corresponding the median volume for LNA scramble and LNA *XACT* are shown, with the *XACT* volume delimited by the dotted white line. (F) Assessment of the chromosome of origin for *Xist* expression related to *XACT* integration at day 4 of differentiation of cells transfected with either scramble or *XACT* LNA, using RNA-FISH for *Xist* (in green) and DNA-FISH for *XACT* (in red). Barplot on the right panel shows the quantification of the respective expression patterns for three independent KD experiments, the error bars corresponding to the SD. Patterns were compared independently for each experiment using a Fisher’s exact test; the p value was always below 10^−4^. The white scale bars in RNA-FISH images represent 5 μm and 10 μm for metaphases.

The dispersed nature of the *XIST* RNA signal on active X chromosomes in human embryos and naive hESCs where it accumulates with *XACT*, together with the lack of co-localization of *XIST* and *XACT*, raises the hypothesis that *XACT* might perturb the tight localization of *XIST* across the chromosome and/or its silencing capacities. To test these hypotheses, we sought to generate a system in which *XACT* expression would precede *XIST* upregulation. For this, we inserted a BAC transgene containing a large part of *XACT* including its promoter region into mouse ESCs. By using first an untargeted approach, we obtained one clone (clone R1) in which insertion of the *XACT* BAC randomly occurred on one of the two Xs, distal to the *Xist* locus, as shown by DNA-FISH on metaphase spreads (Figure 4B). We next used the CRISPR/Cas9 technology to force the insertion of the BAC onto the X, by targeting the integration between the protein-coding genes *Amot* and *Htr2c*, which corresponds to the *XACT* syntenic region on the mouse X chromosome (Figure 4A) (Vallot et al., 2013). Using this strategy, we isolated several clones with transgene insertion on the X, two of which were selected for further investigation (T1 and T9). As the control we used clones in which an unrelated BAC containing the human *FGD1* gene was similarly inserted on the X and clones in which *XACT* was inserted on autosomes (Figure S4A).

*XACT* expression was specifically detected in the clones with *XACT* BAC transgenes, and in most clones *XACT* formed an RNA cloud in a large proportion of cells (Figure 4B; Figure S4A). The copy number of inserted BACs for each of these clones was determined by real-time qPCR (Figure S4E). *Xist* was appropriately upregulated, albeit to variable extents, when transgenic clones were induced to differentiate, while *XACT* expression tended to decrease upon differentiation, similar to what has been observed in humans (Vallot et al., 2013) (Figures S4B and S4C). Within the population of cells in which *XACT* expression was maintained, we observed a significant bias in the choice of the *Xist*-coated chromosome, with *Xist* being preferentially upregulated from the X not carrying *XACT* in the R1 and T9 clones (Figure 4C; Fisher’s exact test, p < 10^−4^). However, in the T1 clone (as in clones in which the *FGD1* BAC was integrated on the X, Figure S4D), *Xist* was able to accumulate similarly on the transgenic and wild-type (WT) X chromosomes (Figure 4C; Figure S4F). Variation in the expression levels of *XACT* is unlikely to account for those differences, as the T1 clone expressed intermediate levels of *XACT* compared to R1 and T9 clones (Figure S4B). However, we found that the nuclear volume occupied by *XACT* was correlated with its ability to bias *Xist* expression (Figure 4D). *XACT* nuclear volume in R1 and T9 clones was in the same range as in naive hESCs and significantly higher than in the T1 clone. The comparison between the T1 and T9 clones is particularly relevant as they shared the same insertion site for *XACT*.

To further investigate the impact of *XACT* RNA on *Xist* accumulation, we knocked down *XACT* in the T9 clone using LNA Gapmers. Successive rounds of knockdown (KD) both prior to and at the onset of differentiation induced a strong decrease in *XACT* RNA levels and in the *XACT* RNA cloud volume (Figure 4E; Figure S4G). By performing simultaneous RNA-DNA-FISH experiments to assess the localization of the *Xist* cloud with respect to the *XACT* transgene (independently of *XACT* expression), we showed that *XACT* KD reverted XCI to random, with *Xist* accumulating in the same proportion on the WT or on the *XACT* transgenic X chromosome (Figure 4F). Altogether, our data demonstrate that robust accumulation of *XACT* on one X chromosome directly impacts the expression or localization of *Xist* in *cis*.

## Discussion

Here, by benchmarking the naive state of pluripotency to the human embryo, we have demonstrated that the pre-XCI state in humans is characterized by the simultaneous accumulation of *XIST* and *XACT* on active X chromosomes. This scenario, which differs radically from the mouse, highlights the plasticity of epigenetic regulations across species and the contribution of lncRNAs to species specificity.

During human development, *XIST* expression and accumulation on X chromosomes begin rapidly after ZGA, yet it takes several divisions before *XIST* initiates XCI. While we confirm here the lack of chromosome-wide XCI prior to implantation, our allelic analysis of RNA-seq data points to an initiation of silencing at the late pre-implantation stages, which would act initially at a gene-to-gene level. We moreover show that active X chromosomes decorated by *XIST* are also characteristic of naive-state pluripotency in vitro. *XIST* expression is, however, highly unstable in this context, suggesting that culture conditions to maintain cells in the naive state are not yet optimum and that *XIST*-positive cells might be counter-selected in current conditions. Bi-allelic *XIST* accumulation might thus be used as a biomarker for defining naive human pluripotency, in the search for refined growth conditions. In addition, during the transition from primed to naive pluripotency, XCR appears to be uncoupled from the major transcriptional and morphological resetting of the cells and likely occurs late in the course of the reprogramming process.

Our findings reveal that *XIST* adopts a peculiar, more dispersed configuration in naive contexts, which may underlie its poor silencing ability. In addition, this scattered accumulation appears not sufficient to recruit repressive histone marks on the X chromosomes. We furthermore show that pre-inactive X chromosomes also accumulate *XACT*, both in vitro and in vivo, demonstrating that *XACT* expression is not restricted to cultured cells and further highlighting its biological relevance. The concomitant activation of *XIST* and *XACT* following fertilization is suggestive of a concerted action in a common biological process.

The facts that *XIST* and *XACT* RNAs co-accumulate yet barely overlap and that introducing *XACT* into a heterologous system influences *Xist* RNA accumulation in *cis* further suggest that *XACT* may act by controlling the association of *XIST* to the chromosome in *cis*, possibly to antagonize or temper its silencing ability. Alternatively, *XACT* could directly participate in the compensatory mechanism occurring at these early stages (Petropoulos et al., 2016), by controlling X chromosome transcriptional outcome. In both cases, this raises the hypothesis that *XACT* function might be linked to the lack of tight control of *XIST* expression in early human development. In this scenario, *XACT* might have evolved to prevent X chromosome silencing and functional nullisomy and to permit an alternative strategy of dosage compensation at these critical developmental stages.

## Experimental Procedures

### Comprehensive Analysis of Single-Cell RNA-Seq Datasets

We gathered single-cell RNA-seq samples from four independent studies on human embryos (Blakeley et al., 2015, Petropoulos et al., 2016, Xue et al., 2013, Yan et al., 2013). We merged together the samples from the three studies (dataset 1 [Yan et al., 2013, Xue et al., 2013, Blakeley et al., 2015], n = 146) where embryos had been classified according their developmental stage, whereas in the last study (dataset 2 [Petropoulos et al., 2016], n = 1,529) embryos were classified according to their last day of in vitro culture.

### Assessment of XCI in RNA-Seq Samples

We used the normalized number of bi-allelic positions on the X chromosome in the RNA-seq datasets as a marker of the activity of the two X chromosomes in female cells. A different number of bi-allelic positions between two conditions is indicative of a change in the transcription balance of two X chromosomes. We also repeated the analysis performed by Petropoulos et al. (2016) to compare with our results.

### Collection of Human Embryos and RNA-FISH

French Biomedecine Agency authorization was obtained for the experimental use of supernumerary cryopreserved embryos resulting from infertility treatment and donated for research (RE 10-032R/RE 12-012R). Written consents were obtained from the couples that their cryopreserved embryos could be used for the research. Human cryopreserved embryos were obtained at Bichat Hospital (Assistance Publique – Hôpitaux de Paris) after in vitro fertilization (IVF) and intra-cytoplasmic sperm injection (ICSI). Day 2–3 cryopreserved embryos were thawed (Embryo Thawing Pack, Origio) and were individually placed in fresh and equilibrated 30 μl culture medium (ISM1 culture medium from day 2 to day 3 and Blastassist from day 3 and on, Origio) at 37°C, 5% CO_2_, under oil and humidified atmosphere. Embryos were cultivated until day 4 (morula) or day 5 and 6 (blastocyst stage). Blastocysts were evaluated using a Leica AM 6000 B inversed microscope at ×400 by an experienced embryologist. An embryo was included in this study only if the embryologist agreed on viability. Blastocysts were classified according to Gardner’s classification (Gardner et al., 2000), taking into account the global morphology and inner cell mass and TE aspect from mid-blastocyst. RNA-FISH was carried out as described previously (Okamoto et al., 2011) (see the Supplemental Experimental Procedures).

## Author Contributions

C.V. and C.R. conceived the project and planned experiments. C.V., C.H., M.C., and M.T. performed the experiments. C.P., N.F., and E.H. provided and contributed to the analysis of human embryos. A.J.C. and P.J.R.-G. provided and performed experiments on naive hESCs. T.M.L.A. and C.V. performed bioinformatic analyses. C.V. and C.R. wrote the manuscript. C.P., A.J.C., and C.H. contributed equally to this work. All authors commented on and revised the manuscript.

## Supplemental Information

Document S1. Supplemental Experimental Procedures and Figures S1–S4

Document S2. Article plus Supplemental Information
