# Supplementary material for: XACT Noncoding RNA Competes with XIST in the Control of X Chromosome Activity during Human Early Development
Source: Cell Stem Cell. 2017 Jan 5;20(1):102–11. doi: 10.1016/j.stem.2016.10.014 (PMC5222720; doi:10.1016/j.stem.2016.10.014)
Supplement: Document S1. Supplemental Experimental Procedures and Figures S1–S4 [file mmc1.pdf]

Cell Stem Cell, Volume *20*

Supplemental Information

XACT Noncoding RNA Competes with *XIST* in the Control of X Chromosome Activity during Human Early Development

Céline Vallot, Catherine Patrat, Amanda J. Collier, Christophe Huret, Miguel Casanova, Tharvesh M. Liyakat Ali, Matteo Tosolini, Nelly Frydman, Edith Heard, Peter J. Rugg-Gunn, and Claire Rougeulle

Supplementary Experimental Procedures

Comprehensive analysis of single-cell RNA-seq datasets

We gathered single-cell RNA-seq samples from four independent studies on human embryos. We gathered single-cell RNA-seq samples from four independent studies on human embryos: GSE36552 (Yan et al., 2013), GSE44183 (Xue et al., 2013), GSE66507 (Blakeley et al., 2015), E-MTAB-3929 (Petropoulos et al., 2016). All reads were aligned to the human genome (hg19) using Tophat2 (Kim et al., 2013) and only uniquely mapped reads were kept. Gene expression levels (Ensembl annotation GRCh37) were estimated as reads (or fragments for paired-end libraries) per kilobase of exon model and per million of mapped reads: RPKM (or FPKM). We first used htseq-count (Anders et al., 2015) to estimate the number of reads mapping to each gene within the reference annotation. Reads overlapping two genes were discarded. We then used an in-house script (R, https://www.r-project.org) to normalize the count matrices by coding length of each gene and by library size. Throughout the paper we used log2(RPKM +0.001) as expression levels for representation, unsupervised analysis and calculus of Spearman's correlation scores. All subsequent analysis were conducted in R. Principal Component analysis for the n=1000 most variant genes of each dataset (genes with the highest standard deviation) was used to represent the two datasets in Fig. 1a. To determine the sex of each cell within datasets #1 and #2 (and subsequently each embryo), we used the sum of the expression of the Y-linked genes (as in Petropoulos et al. (Petropoulos et al., 2016)). Seeing the bimodal distribution of the $\Sigma RPKM_{Ygenes}$ (Supplementary Fig. 1a), cells with $\Sigma RPKM_{Ygenes} < 50$ were classified as female and cells with $\Sigma RPKM_{Ygenes} > 100$ were classified as male. Since the zygotic genome activation occurs between the 4 and 8-cell stage, we could only determine with confidence the sex of cells from a stage later than 4-cell in dataset #1 and starting from E4 in dataset #2.

We used standard R functions to perform hierarchical clustering (with Euclidian distance and average linkage). We used consensus clustering (Bioconductor *ConsensusClusterPlus* package) (Wilkerson and Hayes, 2010) to examine the stability of the clusters. We established consensus partitions of the data set in K clusters (for K = 2, 3 to 10), on the basis of 1 000 resampling iterations (80% of genes, 80% of sample) of hierarchical clustering, with Euclidian distance as the distance metric and average method for linkage analysis. Using the cumulative distribution functions (CDFs) of the consensus matrices and its coupled plot of the relative change in area under the CDF curve (Supplementary Fig. 1c), we observed that a standard partition in k=3 clusters (TE-like, PE-like and EPI-like) was highly unstable, and samples often changed groups when performing repetitive clustering. We chose a partition in k= 4 clusters, named TE, PE, EPI and EPI-TE according to the pattern of expression of the 75-gene signature. We estimated the distribution and variability of *XACT* and *XIST* expression levels in female late stage blastocyst according to lineage, using the median value of log2 expression levels for each lncRNA in TE, PE, EPI and EPI-TE and its associated median absolute deviation (MAD).

Assessment of X-chromosome inactivation in RNA-seq samples

We started from the set of uniquely aligned reads (see above). We filtered out PCR duplicates using Picard tools (http://picard.sourceforge.net). We then applied the GATK pipeline (McKenna et al., 2010) to identify high-confidence positions with bi-allelic expression on the X chromosome (chrX) and on chromosome 7 (chr7) by base quality score recalibration, indel realignment, and SNP

discovery across each individual sample using standard hard filtering parameters to minimize the number of false positives (QUALITY≥100, coverage ≥10, SNP within the dbSNP database (build 137), AF=0.5). We normalized the number of X-linked bi-allelic positions by the number of positions potentially interrogated by our pipeline, *i.e* the number of known SNPs (dbsnp_137.b37) on the chrX with a coverage≥10 reads. We used the number of bi-allelic positions on chr7 as quality metrics of the RNA-seq samples (as an autosome should be bi-allelically expressed in all samples) and kept for further analysis cells with a number of bi-allelic positions on chr7 $n_{chr7}>19$. We represented the normalized number of X-linked bi-allelic positions using boxplots and pooling the samples according to stage (day for dataset #2 and developmental stage dor dataset #1) or to *XACT* expression (negative for log2 < -9 and positive for log2 ≥ -9). We compared the distribution of these numbers between categories using Wilcoxon's rank test. We also compared the numbers of X-linked bi-allelic positions to the numbers of positions found for chr1, chr 2 and chr 7.

We also repeated the analysis performed by Petropoulos *et al.* to compare with our results (Petropoulos et al., 2016). Briefly, we used SAMtools mpileup (Li et al., 2009) to retrieve allelic read counts for positions in dbSNP (build 137) with a minimal coverage of 3 and a threshold for the allelic ratio between the major and minor allele of 10 to separate mono and bi-allelic expressed positions. We computed a 'bi-allelic' ratio between the chrX and chr7, which is a ratio of the fractions of positions with bi-allelic calls over the total number of positions with sufficient coverage for each chromosome (Supplementary Fig. 1g). With a threshold for the allelic ratio of 10, we do not find any statistical difference in these ratios between different stages as in Petropoulos *et al.*. However when we decreased this threshold to 3 (the minor allele with at least 25% of the reads), we find a significant decrease of the number of these positions on the chrX compared to autosome along stage progression. The differences between observations made with an allelic ratio of 10 or 3 indicate that there is an imbalance between the two X chromosomes starting at E6, as there are less positions with equivalent transcription from each X.

RNA-FISH on human embryos

French Biomedecine Agency authorization was obtained for the experimental use of supernumerary cryopreserved embryos resulting from infertility treatment and donated for research (RE 10-032R/RE 12-012R). Written consents were obtained from the couples that their cryopreserved embryos could be used for the research. Briefly, after removal of the *zona pellucida*, embryos were rinsed in embryo culture medium and transferred onto a Denhardt's solution-coated coverslip and air-dried for 30 min at RT. Embryos were then fixed in 3% paraformaldehyde for 10 min at RT and permeabilised on ice in PBS with 0.5% Triton X-100 and 2mM Vanadyl Ribonucleoside Complex (New England Biolabs) for 8 to 12 min depending on the embryo stage, and then progressively dehydrated in ethanol. The coverslips were kept in 70% ethanol at -20°C before RNA-FISH. SpectrumGreen or SpectrumRed-dUTP (Vysis) were used to generate by nick translation labeled probes for *XIST* (a 10kb fragment corresponding to *XIST* exon 1, (gift from Dr. Carolyn Brown, Department of Medical Genetics, University of British Columbia, Vancouver, British Columbia, Canada) and for *XACT* (RP11-35D3, BACPAC). Prior to hybridization, 0.1µg of probe was ethanol-precipitated together with 10 µg of salmon sperm (Thermo Fisher Scientific) for *XIST* and 10µg of human Cot-1 (Thermo Fisher Scientific) for *XACT*, washed twice in 70% ethanol and resuspended in formamide (Sigma-Aldrich). The probes were denaturated at 75°C for 7 min, mixed with an equal quantity of 2x hybridization buffer (4XSSC, 20% Dextran Sulfate, 2mg/ml BSA, 2mM Vanadyl Ribonucleoside Complex (New England Biolabs))

and kept on ice before use. Embryos were hybridized with labeled probes overnight at 37°C in a dark and humid chamber. After three washes in 50% formamide/2x SSC, three washes in 2x SSC at 42°C, coverslips were counterstained with DAPI (1ug/ml), mounted and viewed under the fluorescence microscope. A 200M Axiovert (Zeiss) fluorescence microscope equipped with an ApoTome system was used for image acquisition and the generation of optical sections in 3D. Sequential z-axis images were collected in 0.3µm steps. At the blastocyst stage, when possible, we were able to distinguish cells corresponding to the trophectoderm or the inner cell mass according to their morphological aspect.

Cell culture of primed and naïve human embryonic stem cells

WA09/H9 NK2 and WA01/H1 primed hESC and their naïve counterparts were kindly provided by Austin Smith with permission from WiCell (Takashima et al., 2014). Primed WIBR3 hESC were kindly provided by Rudolph Jaenisch (Theunissen et al., 2014). Primed hESC were cultured on CF1 irradiated mouse embryonic fibroblasts (MEF) in Advanced DMEM containing 20% Knockout Serum Replacement (Thermo Fisher Scientific) supplemented with 2mM L-Glutamine (Thermo Fisher Scientific), 0.1mM β-mercaptoethanol (Sigma-Aldrich), 1x Penicillin/Streptomycin (Thermo Fisher Scientific), 1x Non-Essential Amino Acids (Thermo Fisher Scientific) and 4ng/ml FGF2 (WT-MRC Cambridge Stem Cell Institute). Naïve H9 and H1 hESC were cultured on CF1 MEF in a 1:1 mixture of DMEM/F12 and Neurobasal (Thermo Fisher Scientific), 0.5x N2-supplement (Thermo Fisher Scientific), 0.5x B27-supplement (Thermo Fisher Scientific), 1x Non-Essential Amino Acids (Thermo Fisher Scientific), 2mM L-Glutamine (Thermo Fisher Scientific), 1x Penicillin/Streptomycin (Thermo Fisher Scientific), 0.1mM β-mercaptoethanol (Sigma-Aldrich), 1µM PD0325901, 1µM CHIR99021, 20ng/ml human LIF (all from WT-MRC Cambridge Stem Cell Institute) and 2µM Gö6983 (Sigma-Aldrich). Naïve WIBR3 hESC were converted from primed hESC as follows. Primed hESC were dissociated into single cells with Accutase and seeded at a density of 2x104 cells/cm2 in primed hESC medium containing 10µM Y-27632 on MEF-coated plates. The following day, media was changed to 5iLA, which consists of a 1:1 mixture of DMEM/F12 and Neurobasal (Thermo Fisher Scientific), 1x N2-supplement (Thermo Fisher Scientific), 1x B27-supplement (Thermo Fisher Scientific), 1x Non-Essential Amino Acids (Thermo Fisher Scientific), 2mM L-Glutamine (Thermo Fisher Scientific), 1x Penicillin/Streptomycin (Thermo Fisher Scientific), 0.1mM β-mercaptoethanol (Sigma-Aldrich), 50µg/ml Bovine Serum Albumin (Thermo Fisher Scientific), 1µM PD0325901 (WT-MRC Cambridge Stem Cell Institute), 0.5µM IM-12 (Cell Guidance Systems), 0.5µM SB590885 (Cell Guidance Systems), 1µM WH4-023 (Cell Guidance Systems), 10µM Y-27632 (Cell Guidance Systems), 20ng/ml human LIF (WT-MRC Cambridge Stem Cell Institute) and 10ng/ml Activin A (WT-MRC Cambridge Stem Cell Institute). Cells were passaged after six days with Accutase at a 1:4 split ratio and expanded for several passages in 5iLA media on MEF-coated plates. All cells were cultured in 5% O2, 5% CO2 at 37°C. Authentication of hESC was achieved by confirmation of expression of pluripotency gene and protein markers. Cells were routinely verified as mycoplasma-free using a PCR-based assay. No cell lines used in this study were found in the database of commonly misidentified cell lines that is maintained by ICLAC and NCBI Biosample.

Transitioning primed cells using RSeT

To transition primed cells, we started from human-embryo derived H9 (P40-P52) and WIBR2 (P55-P67) cells, which were maintained in conventional feeder-free PSC culture conditions with MTeSR1™

(STEMCELL technologies) on Matrigel hES-qualified Matrix (Corning) coated plates. For resetting of hESCs into a naïve-like state, cells were cultured in RSeT™ medium, following the guidelines of the manufacturer (STEMCELL technologies). Briefly, hESCs in mTeSR1™ were split with Gentle Dissociation solution (STEMCELL technology) and cell aggregates were plated onto a layer of MEF feeders with MTeSR1™ under normoxic conditions (20% O2, 5%CO2). The following day, the medium was replaced with RSeT™ and cells were further cultivated under hypoxic conditions (5% O2, 5% CO2). Medium was changed daily. Cells were split every 4-6 days, after dissociation with TrypLE express (Thermo Fisher Scientific). After each passage, cells were incubated on RSeT™ in the presence of Rho-associated kinase inhibitor (ROCKi) (Y-27632, EMD Millipore), until the following medium change. Transcriptional changes indicative of pluripotency (Grow et al., 2015; Weinberger et al., 2016) where assessed by qRT-PCR.

Allelic expression analysis of X-linked transcripts in primed and naïve H9 cells

We used published RNA-seq datasets for primed and naïve H9 cells: GSE60945 (Takashima et al., 2014). We took advantage of the clonal XCI pattern of H9 cells (Mitjavila-Garcia et al., 2010; Shen et al., 2008) to analyze the data in an allelic manner. We had previously identified a set of SNPs on the X chromosome (198 SNPs on the X chromosome, corresponding to 78 genes) that could be used for allelic analysis (Vallot et al., 2015). We considered a transcript as bi-allelic when at least 25% of reads were originating from the minor allele (allelic ratio 3:1). We computed the allelic information by gene and counted the number of bi-allelic and mono-allelic expressed gene in both primed and naïve H9 cells and compared them using a Fisher's exact test.

RNA and DNA-FISH on cell lines

Cells were fixed at room temperature in 3% Paraformaldehyde (Electron Microscopy Science)/PBS for 10min and permeabilized as previously described (Vallot et al., 2015) in CSK buffer supplemented with 0.5% Triton (Sigma-Aldrich), 2mM EGTA (Sigma-Aldrich) and 2mM VRC (New England Biolabs) for 5min, and washed 3 times in ice-cold 70% Ethanol. SpectrumGreen or SpectrumRed-labeled probes (Vysis) were generated by nick translation for human *XIST* (see above), mouse *Xist* (p510), *XACT* (RP11-35D3, BACPAC), *ATRX* (RP11-42M11, BACPAC Resource), *FGD1* (RP11-625D4, BACPAC) and *POLA1* (RP11-1104L9, BACPAC). For RNA and DNA-FISH, all probes generated from BACs were precipitated with human Cot-1 DNA (Thermo Fisher Scientific) and the *XIST*-probe with Salmon Sperm DNA (Thermo Fisher Scientific), resuspended in formamide and denatured for 7min at 75°C. Probes are then diluted in an equal volume of 2X Hybridization Buffer (4XSSC, 20% Dextran Sulfate, 2mg/ml BSA, 2mM Vanadyl Ribonucleoside Complex). Cot-1 precipitated probes are additionally pre-incubated 15min at 37°C.

For RNA-FISH, coverslips were dehydrated in 90% and 100% ethanol and incubated overnight with probe at 37°C. After three 50% formaldehyde/2XSSC washes and three 2XSSC washes at 42°C for 4min, coverslips were mounted in Vectashield plus DAPI (Vector Laboratories).

For DNA-FISH, coverslips were dehydrated in 70%, 90% and 100% ethanol and adapted in 2XSCC at 80°C. Coverslips were denaturated for 10min at 80°C in 70% formamide/2XSSC (pH=7.2) and were dehydrated in 70%, 90% and 100% ethanol and incubated overnight with probe at 37°C. After three 2XSSC washes at 45°C and three 0.1XSSC washes at 60°C for 4min, coverslips were mounted in Vectashield plus DAPI.

Microscopy and image analysis

All images were taken on a fluorescence DMI-6000 inverted microscope with a motorized stage (Leica) and with a CCD Camera HQ2 (Roper Scientifics) controlled by the Metamorph 7.04 software (Roper Scientifics) using a HCX PL APO 100X oil objective (numerical aperture, 1.4, Leica). Optical Z-sections were collected at 0.5µm steps through each nucleus at different wavelengths depending on the probes used (DAPI [360nm, 470nm], FITC [470nm, 525nm], cy3 [550nm, 570nm], Texas Red [596nm, 612nm] and cy5 [647nm, 668nm]); approximately 30 optical sections per nucleus were collected. Stacks were processed using ImageJ 1.46 (Abramoff et al., 2004), and throughout the manuscript the 3D-FISH experiments are represented as a 2D-projection of the stacks (maximum projection).

We designed in-house ImageJ macros to extract quantitative information from 3D-images and perform subsequent image analysis in R. We automatically extracted from each nucleus the fluorescence value of each pixel from *XACT* and *XIST* signal, as well as Yen's threshold for each signal. In R, we imported the matrices of fluorescence values and their associated thresholds, and limited our analysis to pixels above respective thresholds for *XACT* and *XIST* images. We first converted the positions of pixels in µm, according to the camera and objective settings. We computed the dispersion of *XIST* RNA, by comparing the cumulative volume of the signal to the theoretical spherical volume it could occupy based on the maximal radial distance (Fig. 3G). We computed the cumulative volume occupied by *XACT* RNA, by adding the volume of all pixels with intensity above threshold. To compare the localization of *XACT* and *XIST* RNAs, for each image (corresponding to an individual X chromosome), we compared the intensity of fluorescence on the union of pixels with signal above threshold for *XACT* or *XIST* signal through the calculus of a Spearman's correlation score and p-value for at least n=90 images.

Immunofluorescence and RNA-FISH

Naïve cells were cultured on 12mm coverslips and were first fixed in 3% Paraformaldehyde/PBS for 10min at RT and then permeabilized for 5min at RT with CSK buffer (NaCl, MgCl2, Sucrose, PIPES, pH=6.8) supplemented with 0.5% Triton and 2mM EGTA. Cells were incubated 45min with 0.2% Gelatin/PBS, 45min with rabbit polyclonal primary antibody (anti-H3K4me2 (ab7766 Abcam), anti-H3K27me3 (07-449 Millipore), anti-H3K9me3 (gift from from Pr. Prim Singh (Cowell et al., 2002) and anti-H3K27Ac (ab4729 Abcam)) diluted in 0.2% Gelatin/PBS, washed 3 times in PBS, incubated 30min with an Alexa Fluor 488nm anti-rabbit secondary antibody (Life Technologies) and washed 3 times with PBS. Cells were fixed in 3% Paraformaldehyde/PBS for 10min at RT before proceeding to RNA-FISH as described above. Immunofluorescence and RNA-FISH signals were simultaneously observed using a confocal microscope DMI6000 TCS SP5 (Leica). To evaluate the colocalization between *XACT*, *XIST* and histone marks, we compared grey values along line scans within single z-sections.

Analysis of ChIP-seq datasets

We used our previously published H3K27me3 and H3K9me3 ChIP-seq datasets for primed *XIST*+ hESCs (GSE62562) as well as published H3K27me3 and H3K9me3 ChIP-seq datasets for naïve *XIST*+ hESCs (GSE59435 and GSE84382). We analysed fastq files as previously described (Vallot et al., 2015). We subdivided the X chromosome in 100kbp windows, and computed for each window the number of reads from the ChIP and the input experiment. For the scatterplots, we defined the enrichment as

the ratio of the number of reads in the ChIP over the input in these 100kbp-windows. We compared ratios between cell lines using Spearman's correlation scores.

Generation of mouse ES cells with *XACT* or *FGD1* human genes

Female LF2 ES cell lines were grown in Dulbecco's modified Eagle medium (DMEM, Thermo Fisher Scientific), 15% Fetal calf serum (FCS, Thermo Fisher Scientific) and 1000 U/mL LIF (Millipore), on gelatin-coated dishes and in absence of feeder cells. Cell passaging (1:6 split) was done by enzymatic treatment with trypsin (Trypsin-EDTA 0.05%, Thermo Fisher Scientific) for 4 minutes. Cells were transfected using Lipofectamine2000 (Thermo Fisher Scientific). For random BAC integration, cells were transfected with a BAC encompassing part of the *XACT* locus including its promoter (RP11-110P23, chrX: 113085338- 113248350) and a plasmid encoding a Neomycin resistance gene (ratio 1:3). For targeted integration, LF2 cells were co-transfected with a *XACT* BAC (or *FGD1* BAC RP11-625D4) together with a plasmid (pSpCas9(BB)-2A-GFP (PX458), gift from Feng Zhang (Addgene plasmid # 48138)) containing the Cas9 gene, and a gRNA. We used two gRNAs, designed with CRIPSR Design (http://crispr.mit.edu), targeting the mouse region syntenic to the human *XACT* locus; the first one targeting chrX: 146088618-146088637, and the second one targeting chrX: 146096270-146096289. Twelve hours post-transfection, cells were plated in 10-cm dishes and left under 0.25µg/ml G418 selection (Thermo Fisher Scientific) for 10 days. Clones were screened for *XACT* or FGD1 insertion by PCR and DNA-FISH, and for *XACT* or *FGD1* expression by RNA-FISH. We determined the copy number of the BAC in each clone, using quantitative real-time PCR. We diluted pure BAC DNA within LF2 genomic DNA to achieve artificial preparations with various ratios of the BAC copy number versus the genomic DNA (100 copies to 1 copy). We normalized the BAC copy number to the copy number of the *Chic1* gene to account for variations in the measurement of the DNA concentrations of the samples.

Differentiation of mouse ES clones

Differentiation experiments were carried out on gelatin-coated dishes at a density of 10 000 cells per cm2, in Dulbecco modified Eagle medium (DMEM, Thermo Fisher Scientific) and 10% Fetal calf serum (FCS, Thermo Fisher Scientific). RNAs were extracted at day 0, 2, 4 and 7 of differentiation from biological triplicates (independent differentiation experiments). Cells were fixed at day 0, 2, 4 and 7 for RNA-FISH.

Knockdown using LNA Gapmers

We designed three different Locked Nucleic Acid (LNA) Gapmers targeting *XACT* and a control scramble LNA Gapmer devoid of target RNA using the Exiqon online tool (Exiqon). LNAs were delivered at 50nM via lipofection using the transfection reagent RNAimax (Invitrogen), according to manufacturer recommendations. We kept for further analysis of the T9_XACT clone, the only LNA Gapmer ('LNA_XACT') and the scrambled Gapmer ('LNA_SCR') that triggered an efficient knockdown of the XACT transgene at day 2 after transfection. We transfected the T9_XACT clone two days prior to the initiation of differentiation (d-2), the day of the differentiation (d0) and two days after the initiation of differentiation (d2). We collected cells for RNA and RNA-DNA FISH experiments at day -2, day 0, day 2 and day 4, for three independent experiments.

Metaphase spreads and DNA-FISH on mouse clones

Metaphase spreads were prepared as described (Naim et al., 2013). Spreads were then fixed in 0.25% PFA for 10min at RT, dehydrated and denatured 5min at 75°C in 70% Formamide/2X SSC. Mouse X chromosome paint (Metasystems) was denatured 2min at 75°C and applied on slides.

RNA extraction and RT-qPCR

Total RNA was extracted from all cells using trizol (Thermo Fisher Scientific). RNA was treated with Dnase I (Roche) to remove DNA contamination. One µg of total RNA was used for reverse transcription, using the SuperScript II kit (Thermo Fisher Scientific). mRNA expression levels were evaluated using real-time quantitative PCR (RT-qPCR) with the SYBR Green kit on an ABI PRISM 7500 real-time thermal cycler (Applied Biosystems). All samples were run in duplicate. RNA expression levels for the transcripts of interest were normalized against the reference gene *Arpo* for mouse samples and to *U6* for human samples, according to the $2^{-\Delta Ct}$ method.

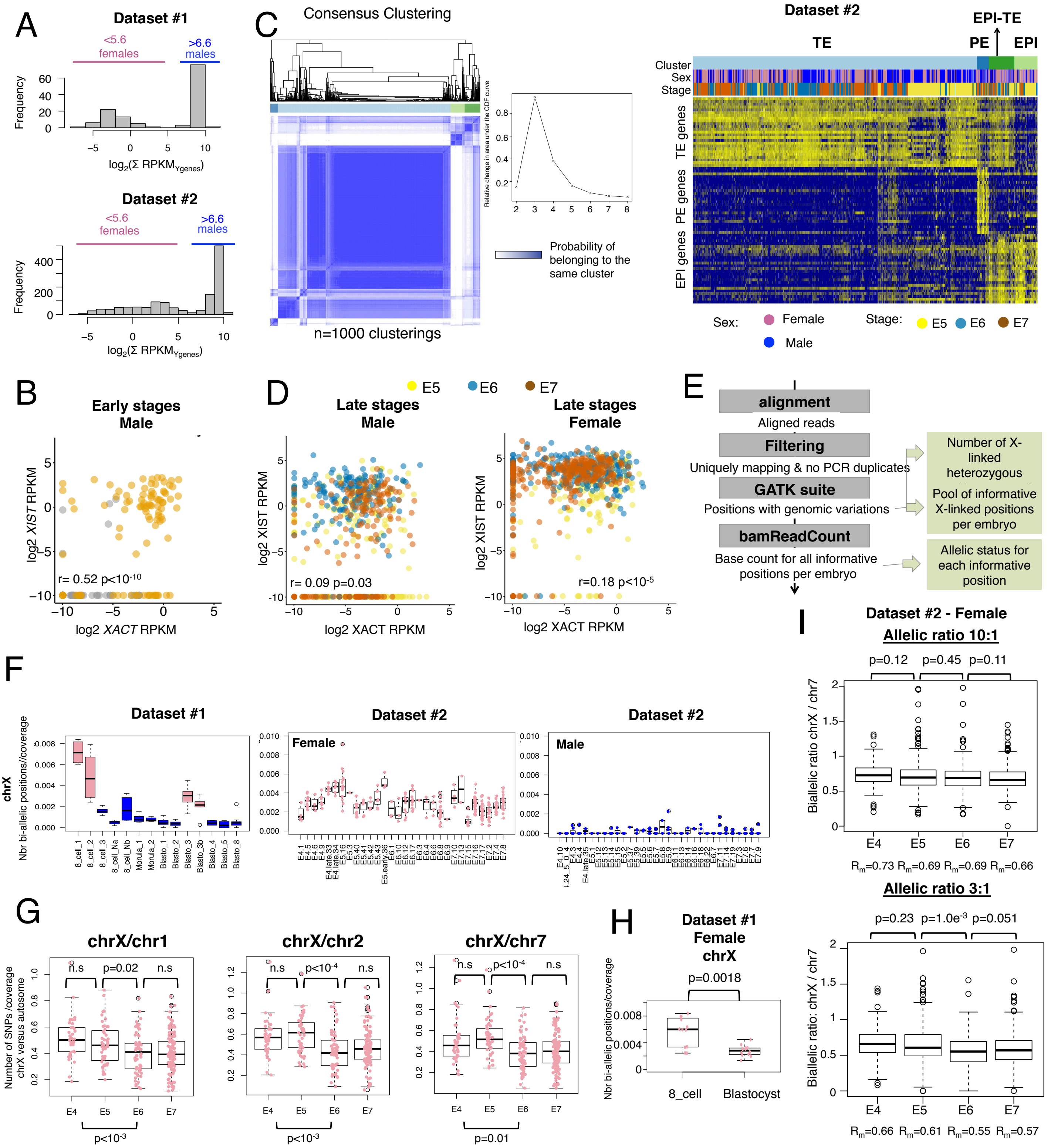

Figure S1, related to Figure 1: **Linking *XACT* and *XIST* expression levels to sex, lineage commitment and X chromosome inactivation in the early steps of human development**.

(**A**) Determination of the sex of each embryo for datasets #1 and 2 as in Petropoulos *et al.* [2]; the histograms display the bimodal distribution of the sum of the RPKM of Y-linked genes for each cell. Cells with $\log2(\Sigma RPKM_{Ygenes})>6.6$ were assigned as males, whereas cells with $\log2(\Sigma RPKM_{Ygenes})<5.6$ were assigned as females. (**B**) Plot of *XACT* versus *XIST* expression levels (log2 RPKM) in early stages male cells of dataset #2 (E3, E4 and early E5), with corresponding Spearman's correlation score and p-value. (**C**) Consensus clustering approach to assess lineage commitment within cells from E5, E6 and E7 embryos. Left panel: Consensus clustering (for k=4 clusters) of all cells (n=1000 hierarchical clustering were performed on 80% randomly chosen samples and 80% randomly chosen genes to evaluate the stability of the clustering) and plot of the area under the CDF curve for each choice of number of cluster (k=2 to 8). The relative change in area does not reach a plateau while k is increasing, indicating that the signature does not permit a fully stable partition of the samples. We chose to partition the samples in k=4 clusters, considering that the corresponding consensus clustering displays a stable partition for PE and part of the EPI and TE samples, moreover k>4 does not resolve the proper partition of samples with a mixed signature. Right panel: Heatmap illustrating the expression pattern of the 75-gene signature (25 genes characterizing each lineage, TE, EPI and PE) and the repartition of the late stage samples into 4 clusters. Each cluster was named according to the expression pattern of the genes characteristic of each lineage. (**D**) Plots of *XACT* versus *XIST* expression levels (log2 RPKM) in late stages male cells of dataset #2 (E5, E6 and E7), with corresponding Spearman's correlation score and p-value. The color code illustrates the stage in the left panel, and the commitment lineage in the right panels (see (c)). (**E**) Schematic summary of the pipeline used to probe X chromosome inactivation in both datasets. Grey boxes relate to tools whereas green boxes relate to results. (**F**) The boxplots represent for each dataset the normalized number of X-linked bi-allelic positions detected per cell grouped per embryo. Each points within the boxplots corresponds to a single cell of the given embryo (pink for females and blue for males). (**G**) Same analysis as in Fig.1E, the numbers of X-linked bi-allelic positions are compared to the numbers for different autosomes (chr1, chr2 and chr7), n.s stands for '*not significant*' (p>0.05). (**H**) Same as in Fig.1E for female samples of dataset #1. (**I**) Computation of the number of bi-allelic positions using a different pipeline (Petropoulos *et al.* 2016), based on the counting across known SNPs on the X-chromosome; boxplots represent the distribution of chr X to chr 7 ratio of normalized number of bi-allelic positions across days of development for an allelic ratio of 10:1 (upper panel) as in Petropoulos et al. 2016 and an allelic ratio of 3:1 (lower panel). Comparisons of distributions within stages were quantified using Wilcoxon's [illegible]

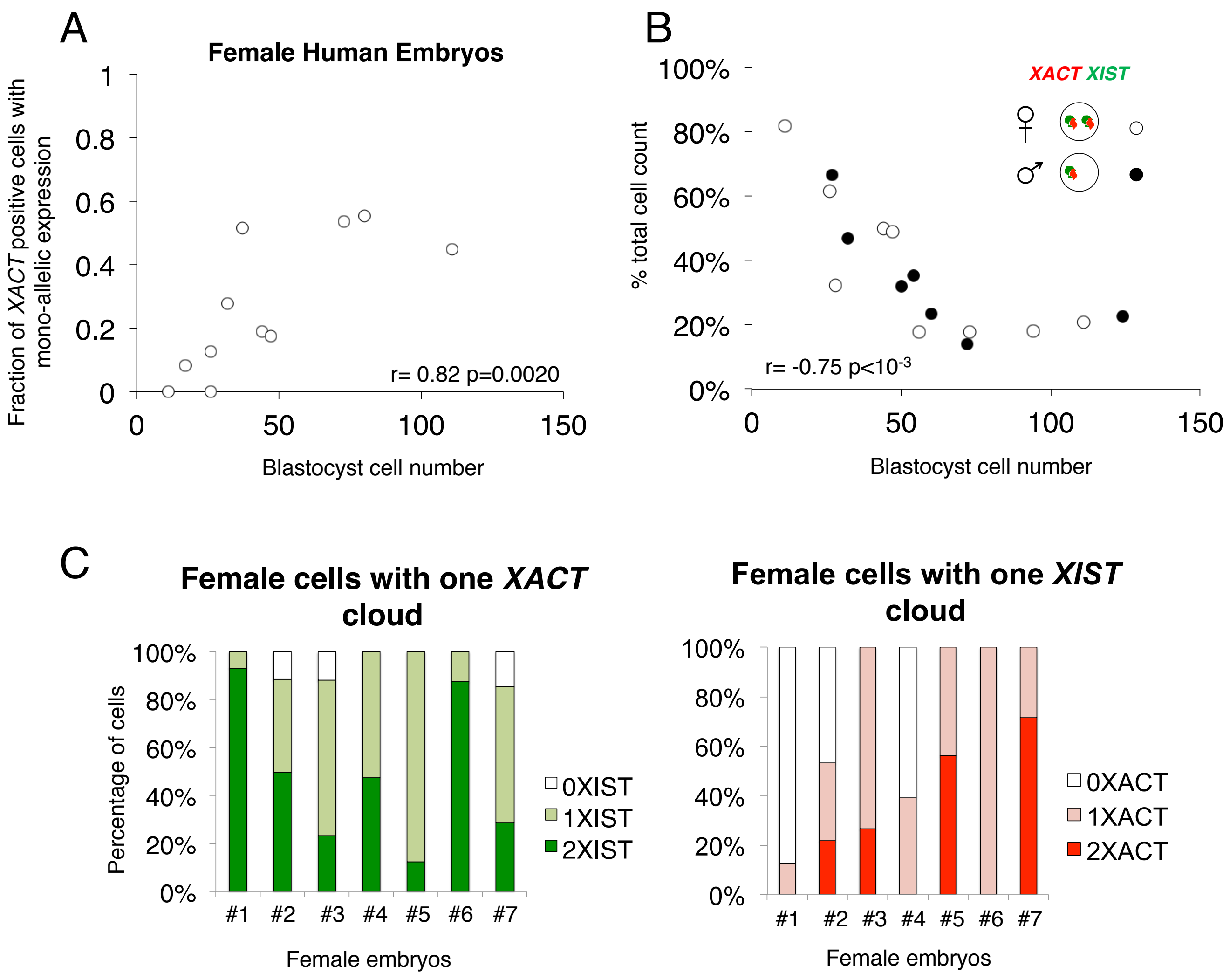

Figure S2, related to Figure 2: ***XIST* and *XACT* expression pattern in human blastocysts.**
(A) The plot represents the fraction of *XACT* positive female cells showing mono-allelic accumulation, according to the blastocyst cell number. **(B)** Plot of the percentage of cells accumulating *XIST* and *XACT* RNAs versus the blastocyst cell number per male (dark circles) and female (white circles) embryos. The correlation between measures was assessed in both cases using a Spearman's correlation test. (**C**) Histograms representing for each female embryo the expression pattern of *XIST* in cells with mono-allelic expression of *XACT*. Same representation for the expression pattern of *XACT* in cells with mono-allelic expression of *XIST*.

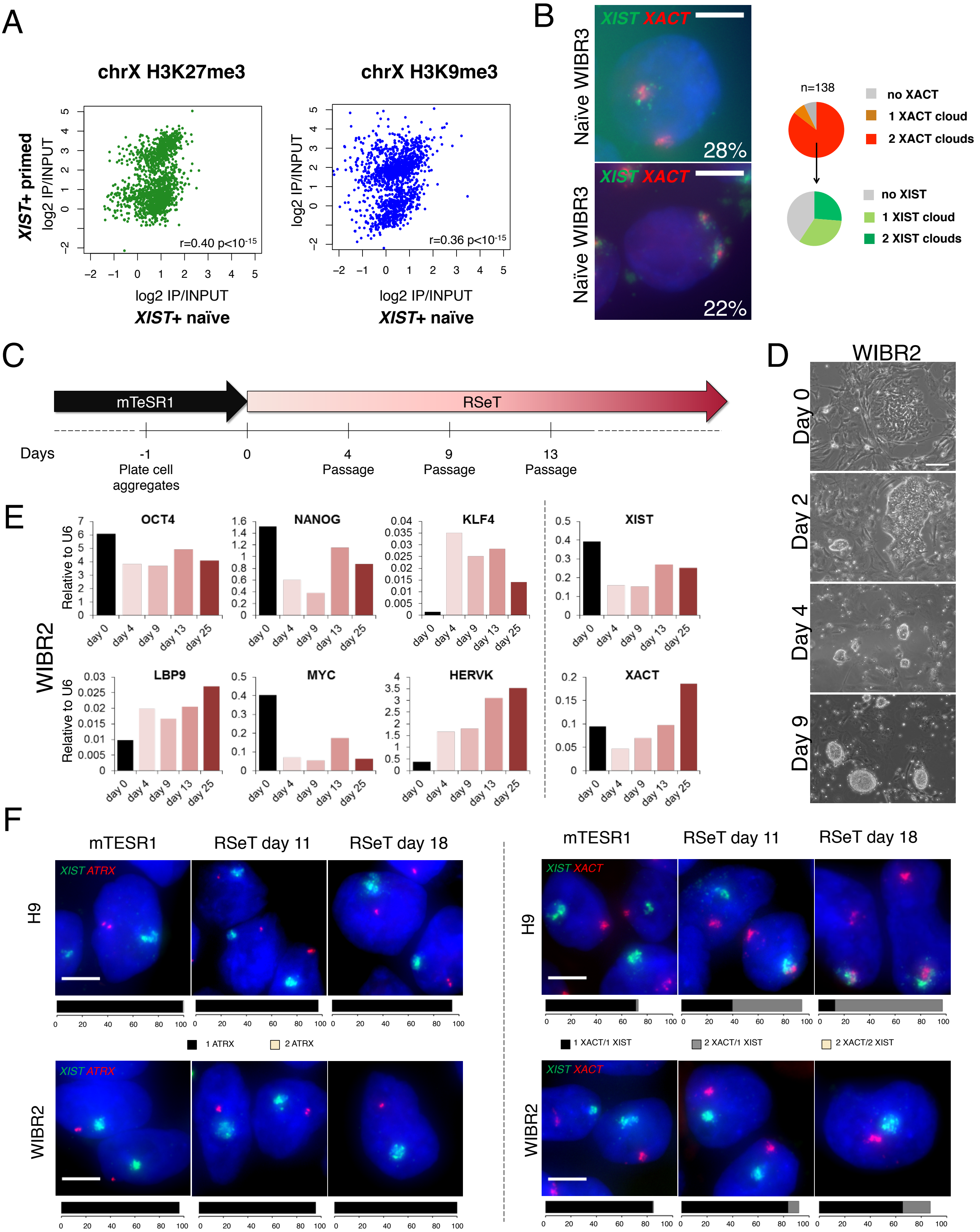

Figure S3, related to Figure 3: **Additional characterization of reprogrammed hESCs. (A)** Scatter plot comparing H3K27me3 and H3K9me3 log2 enrichment ratios on the X chromosome between *XIST*-expressing primed hESCs (H9 (Vallot et al., 2015)) and naive hESCs (WIBR2 for H3K27me3 (Theunissen et al., 2014) and WIBR3 for H3K9me3 (Theunissen et al., 2016)). Log2 ratios were compared using Pearson correlation tests and the associated p-value was calculated using random permutations of the data sets. **(B)** RNA-FISH analysis revealing *XACT* (red) and *XIST* (green) expression pattern in naïve female WIBR3. (**C**) Schematic view of the procedure to transition hESCs from mTeSR to RSeT, H9 and WIBR2. (**D**) Representative bright field images of WIBR2 cells at days 0, 2, 4 and 9 of the transition. (**E**) Left panel: qRT-PCR quantification of markers of naïve and primed pluripotency during the transition of WIBR2 from mTeSR to RSeT. The histograms display the enrichment compared to U6. Right panel: qRT-PCR analysis for *XIST* and *XACT.* (**F**) Left panel: RNA-FISH analysis with *ATRX* (red) and *XIST* (green) probes. Displayed pictures correspond to the most frequent expression pattern at each day of RSeT transition. The barplots beneath the pictures represent the percentage of cells with each expression pattern. Right panel: RNA-FISH analysis with *XACT* (red) and *XIST* (green) probes. The white scale bar corresponds to 5 μm.

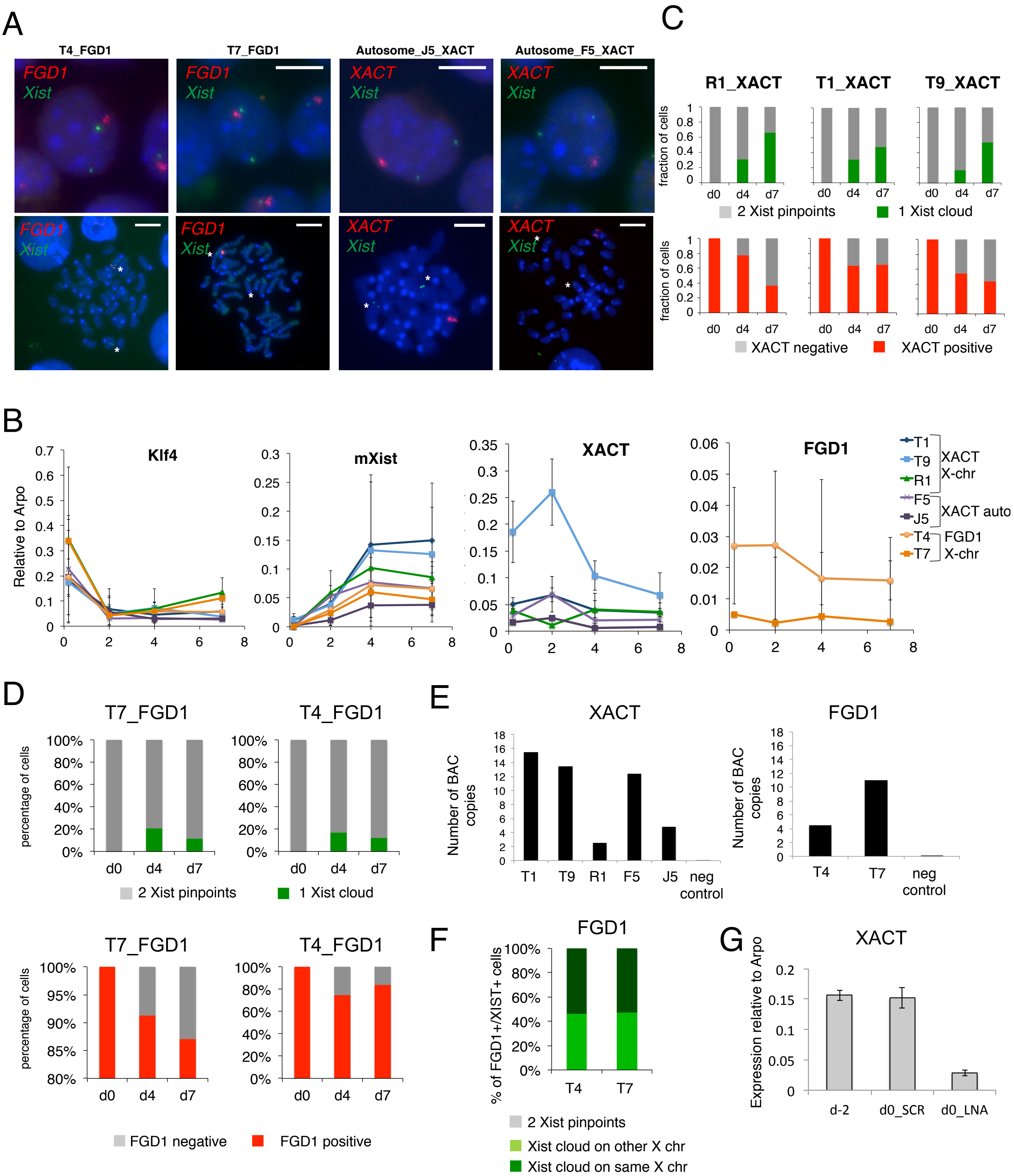

Figure S4, related to Figure 4: **Insertion of human *XACT* or *FGD1* transgenes in mESCs**.
(**A**) Assessment by RNA-FISH of *Xist* (in green) and transgene *(FGD1* or *XACT,* in red*)* expression in interphase nuclei of undifferentiated mouse ES cells (upper panels) and of their localization on metaphase chromosomes (lower panels) in clones with targeted (T4 and T7) integration of *FGD1* and autosomal integration of *XACT* (F5 and J5). The white scale bars correspond to 5μm for interphase nuclei and to 10 μm for metaphases. (**B**) qRT-PCR analysis of *Klf4*, *Xist*, *FGD1* and *XACT* expression in differentiating R1, T1, T9, T4, T7, J5 and F5 clones. (**C**) Quantification of *Xist* and *XACT* RNA-FISH patterns in R1, T1 and T9 *XACT*-transgenic clones. (**D**) Quantification of *Xist* and *FGD1* RNA-FISH patterns in T7 and T4 *FGD1*-transgenic clones. (**E**) The barplot represents the number of BAC copies per cells for *XACT* (left panel) and *FGD1* (right panel), as assessed by qPCR using as a reference serial dilutions of the BAC DNA within LF2 ES cells DNA, and adjusting for differences in DNA concentration with *Chic1* copy number. (**F**) Assessment of the chromosome of origin for *Xist* expression related to *FGD1* integration. In T4 and T7 clones, *Xist* is equally up-regulated from the WT or the transgenic X. A Fisher's exact test was used to compare numbers of nuclei with *Xist* accumulation on WT or transgenic X to a 50/50 repartition. (**G**) Quantification of *XACT* expression using RT-qPCR for 3 independent KD experiments using control LNA Gapmer (SCR) and the LNA Gapmer targeting XACT (LNA) in the T9 clone. Expression is calculated relatively to *Arpo*.

Supplementary references

Abramoff, M.D., Magalhaes, P.J., and Ram, S.J. (2004). Image Processing with ImageJ. Biophotonics International *11*, 36-42.

Anders, S., Pyl, P.T., and Huber, W. (2015). HTSeq--a Python framework to work with high-throughput sequencing data. Bioinformatics *31*, 166-169.

Blakeley, P., Fogarty, N.M., del Valle, I., Wamaitha, S.E., Hu, T.X., Elder, K., Snell, P., Christie, L., Robson, P., and Niakan, K.K. (2015). Defining the three cell lineages of the human blastocyst by single-cell RNA-seq. Development *142*, 3151-3165.

Cowell, I.G., Aucott, R., Mahadevaiah, S.K., Burgoyne, P.S., Huskisson, N., Bongiorni, S., Prantera, G., Fanti, L., Pimpinelli, S., Wu, R., *et al.* (2002). Heterochromatin, HP1 and methylation at lysine 9 of histone H3 in animals. Chromosoma *111*, 22-36.

Grow, E.J., Flynn, R.A., Chavez, S.L., Bayless, N.L., Wossidlo, M., Wesche, D.J., Martin, L., Ware, C.B., Blish, C.A., Chang, H.Y., *et al.* (2015). Intrinsic retroviral reactivation in human preimplantation embryos and pluripotent cells. Nature *522*, 221-225.

Kim, D., Pertea, G., Trapnell, C., Pimentel, H., Kelley, R., and Salzberg, S.L. (2013). TopHat2: accurate alignment of transcriptomes in the presence of insertions, deletions and gene fusions. Genome Biol *14*, R36.

Li, H., Handsaker, B., Wysoker, A., Fennell, T., Ruan, J., Homer, N., Marth, G., Abecasis, G., and Durbin, R. (2009). The Sequence Alignment/Map format and SAMtools. Bioinformatics *25*, 2078-2079.

McKenna, A., Hanna, M., Banks, E., Sivachenko, A., Cibulskis, K., Kernytsky, A., Garimella, K., Altshuler, D., Gabriel, S., Daly, M., *et al.* (2010). The Genome Analysis Toolkit: a MapReduce framework for analyzing next-generation DNA sequencing data. Genome Res *20*, 1297-1303.

Mitjavila-Garcia, M.T., Bonnet, M.L., Yates, F., Haddad, R., Oudrhiri, N., Feraud, O., Magniez, A., Makhlouf, M., Vallot, C., Rougeulle, C., *et al.* (2010). Partial reversal of the methylation pattern of the X-linked gene HUMARA during hematopoietic differentiation of human embryonic stem cells. J Mol Cell Biol *2*, 291-298.

Petropoulos, S., Edsgard, D., Reinius, B., Deng, Q., Panula, S.P., Codeluppi, S., Plaza Reyes, A., Linnarsson, S., Sandberg, R., and Lanner, F. (2016). Single-Cell RNA-Seq Reveals Lineage and X Chromosome Dynamics in Human Preimplantation Embryos. Cell *165*, 1012-1026.

Shen, Y., Matsuno, Y., Fouse, S.D., Rao, N., Root, S., Xu, R., Pellegrini, M., Riggs, A.D., and Fan, G. (2008). X-inactivation in female human embryonic stem cells is in a nonrandom pattern and prone to epigenetic alterations. Proc Natl Acad Sci U S A *105*, 4709-4714.

Takashima, Y., Guo, G., Loos, R., Nichols, J., Ficz, G., Krueger, F., Oxley, D., Santos, F., Clarke, J., Mansfield, W., *et al.* (2014). Resetting transcription factor control circuitry toward ground-state pluripotency in human. Cell *158*, 1254-1269.

Theunissen, T.W., Powell, B.E., Wang, H., Mitalipova, M., Faddah, D.A., Reddy, J., Fan, Z.P., Maetzel, D., Ganz, K., Shi, L., *et al.* (2014). Systematic identification of culture conditions for induction and maintenance of naive human pluripotency. Cell Stem Cell *15*, 471-487.

Vallot, C., Ouimette, J.F., Makhlouf, M., Féraud, O., Pontis, J., Côme, J., Martinat, C., Bennaceur-Griscelli, A., Lalande, M., and Rougeulle, C. (2015). Erosion of X Chromosome Inactivation in Human Pluripotent Cells Initiates with XACT Coating and Depends on a Specific Heterochromatin Landscape. Cell Stem Cell *16*, 533-546.

Weinberger, L., Ayyash, M., Novershtern, N., and Hanna, J.H. (2016). Dynamic stem cell states: naive to primed pluripotency in rodents and humans. Nat Rev Mol Cell Biol *17*, 155-169.

Wilkerson, M.D., and Hayes, D.N. (2010). ConsensusClusterPlus: a class discovery tool with confidence assessments and item tracking. Bioinformatics *26*, 1572-1573.

Xue, Z., Huang, K., Cai, C., Cai, L., Jiang, C.Y., Feng, Y., Liu, Z., Zeng, Q., Cheng, L., Sun, Y.E., *et al.* (2013). Genetic programs in human and mouse early embryos revealed by single-cell RNA sequencing. Nature *500*, 593-597.

Yan, L., Yang, M., Guo, H., Yang, L., Wu, J., Li, R., Liu, P., Lian, Y., Zheng, X., Yan, J., *et al.* (2013). Single-cell RNA-Seq profiling of human preimplantation embryos and embryonic stem cells. Nature structural & molecular biology *20*, 1131-1139.
