# Supplementary material for: XACT Noncoding RNA Competes with XIST in the Control of X Chromosome Activity during Human Early Development
Source: Cell Stem Cell. 2017 Jan 5;20(1):102–11. doi: 10.1016/j.stem.2016.10.014 (PMC5222720; doi:10.1016/j.stem.2016.10.014)
Supplement: Document S2. Article plus Supplemental Information [file mmc2.pdf]

Short Article

Cell Stem Cell

XACT Noncoding RNA Competes with *XIST* in the Control of X Chromosome Activity during Human Early Development

Graphical Abstract

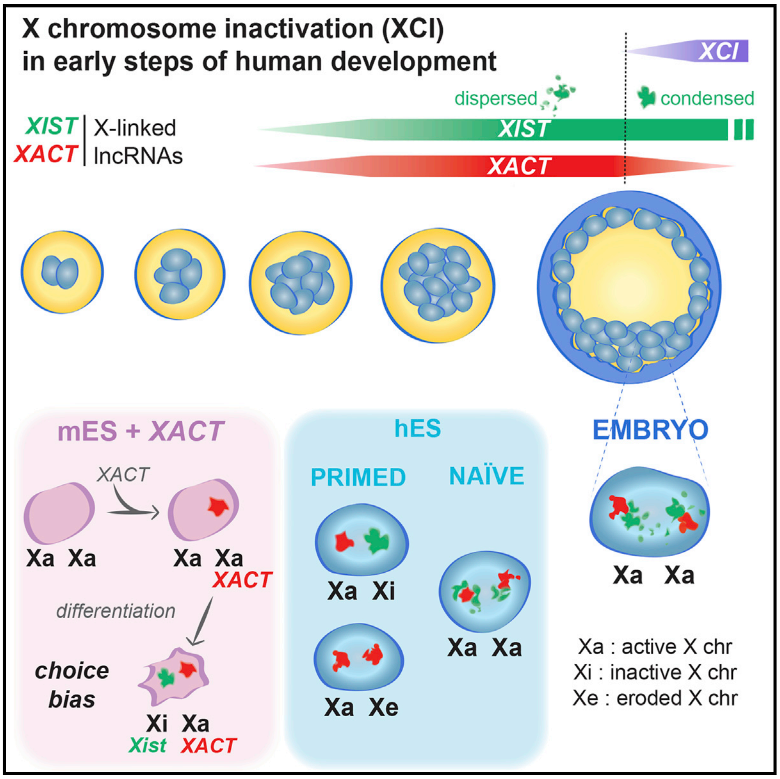

Highlights

- *XACT* and *XIST* simultaneously coat active X chromosomes in human embryos
- Naive hESCs capture the pre-inactive state with *XIST* and *XACT* co-accumulation
- *XIST* adopts a dispersed configuration in pre-inactive cells in vitro and in vivo
- *XACT* prevents *XIST* accumulation in *cis* in a mouse heterologous system

Authors

Céline Vallot, Catherine Patrat, Amanda J. Collier, ..., Edith Heard, Peter J. Rugg-Gunn, Claire Rougeulle

Correspondence

celine.vallot@univ-paris-diderot.fr (C.V.),
claire.rougeulle@univ-paris-diderot.fr (C.R.)

In Brief

Rougeulle and colleagues show that, in human pre-implantation embryos and naive human embryonic stem cells, the lncRNAs *XIST* and *XACT* accumulate together on active X chromosomes. Functional data suggest that *XACT* restrains *XIST* activity before the initiation of X inactivation takes place.

Vallot et al., 2017, Cell Stem Cell *20*, 102–111
January 5, 2017 © 2017 The Authors. Published by Elsevier Inc.
http://dx.doi.org/10.1016/j.stem.2016.10.014

Cell Stem Cell
Short Article

XACT Noncoding RNA Competes with *XIST* in the Control of X Chromosome Activity during Human Early Development

Céline Vallot,[1,*] Catherine Patrat,[2,3,4] Amanda J. Collier,[5,6] Christophe Huret,[1] Miguel Casanova,[1] Tharvesh M. Liyakat Ali,[1] Matteo Tosolini,[1,10] Nelly Frydman,[8,9] Edith Heard,[2] Peter J. Rugg-Gunn,[5,6,7] and Claire Rougeulle[1,11,*]

[1]Sorbonne Paris Cité, Epigenetics and Cell Fate, UMR 7216 Centre National de la Recherche Scientifique (CNRS), Université Paris Diderot, 75013 Paris, France
[2]Mammalian Developmental Epigenetics Group, Institut Curie, PSL Research University, CNRS UMR3215, INSERM U934, 75005 Paris, France
[3]Université Paris Diderot, Sorbonne Paris Cité, 75018 Paris, France
[4]Reproductive Biology Department, AP-HP, Bichat-Claude Bernard Hospital, 75018 Paris, France
[5]Epigenetics Programme, The Babraham Institute, Cambridge CB22 3AT, UK
[6]Wellcome Trust – Medical Research Council Cambridge Stem Cell Institute, University of Cambridge, Cambridge CB2 1QR, UK
[7]Centre for Trophoblast Research, University of Cambridge, Cambridge CB2 3EG, UK
[8]Université Paris-Sud, Clamart 92140, France
[9]Unit of Reproductive Biology, AP-HP, Hôpital Antoine Béclère, Clamart 92141, France
[10]Present address: INRA, UMR1198 Biologie du Développement et Reproduction, 78350 Jouy-en-Josas, France
[11]Lead Contact
*Correspondence: celine.vallot@univ-paris-diderot.fr (C.V.), claire.rougeulle@univ-paris-diderot.fr (C.R.)
http://dx.doi.org/10.1016/j.stem.2016.10.014

SUMMARY

Sex chromosome dosage compensation is essential in most metazoans, but the developmental timing and underlying mechanisms vary significantly, even among placental mammals. Here we identify human-specific mechanisms regulating X chromosome activity in early embryonic development. Single-cell RNA sequencing and imaging revealed co-activation and accumulation of the long noncoding RNAs (lncRNAs) *XACT* and *XIST* on active X chromosomes in both early human pre-implantation embryos and naive human embryonic stem cells. In these contexts, the *XIST* RNA adopts an unusual, highly dispersed organization, which may explain why it does not trigger X chromosome inactivation at this stage. Functional studies in transgenic mouse cells show that *XACT* influences *XIST* accumulation in *cis*. Our findings therefore suggest a mechanism involving antagonistic activity of *XIST* and *XACT* in controlling X chromosome activity in early human embryos, and they highlight the contribution of rapidly evolving lncRNAs to species-specific developmental mechanisms.

INTRODUCTION

In mammals, the activity of the X chromosomes has to be tightly controlled to accommodate the disequilibrium of X-linked gene dosage between males and females. This is achieved through compensatory mechanisms, which serve to equalize X chromosome expression between sexes and relative to autosomes (Disteche, 2016). While X chromosome dosage compensation is essential for proper development, the underlying strategies vary extensively between species. In the mouse, for instance, X chromosome inactivation (XCI) is initiated rapidly after zygotic genome activation (ZGA), several divisions prior to blastocyst implantation. In contrast, human pre-implantation development proceeds in the apparent absence of XCI; instead, dosage compensation is transiently achieved by reducing the expression of both X chromosomes in females (Petropoulos et al., 2016). The mechanisms underlying these differences are still poorly understood.

XCI is triggered by the accumulation of the long noncoding RNA (lncRNA) *XIST* on the chromosome, which recruits protein complexes involved in chromatin remodeling, chromosome structuration, and nuclear organization (Chu et al., 2015; McHugh et al., 2015; Minajigi et al., 2015). Strikingly, however, in human pre-implantation embryos, *XIST* accumulates on every X chromosome in both males and females without inducing robust transcriptional repression or enrichment of H3K27me3, a hallmark of the inactive state (Okamoto et al., 2011). This unusual configuration suggests the existence, in embryos, of mechanisms preventing *XIST*-mediated XCI, which would to some extent be specific to the human, since in the mouse *Xist* accumulation systematically results in the inactivation of the coated chromosome.

We recently have identified *XACT*, an X-linked lncRNA that coats active X chromosomes in human pluripotent stem cells. *XACT* is weakly conserved across mammals and is absent from the mouse, suggesting that it could fulfill a primate-specific function (Vallot et al., 2013). Insight into such function came from the analysis of XCI status and its instability in human embryonic stem cells (hESCs). XCI has indeed been established in most hESCs derived so far, but this status is unstable and hallmarks

 © 2017 The Authors. Published by Elsevier Inc.
This is an open access article under the CC BY license (http://creativecommons.org/licenses/by/4.0/).

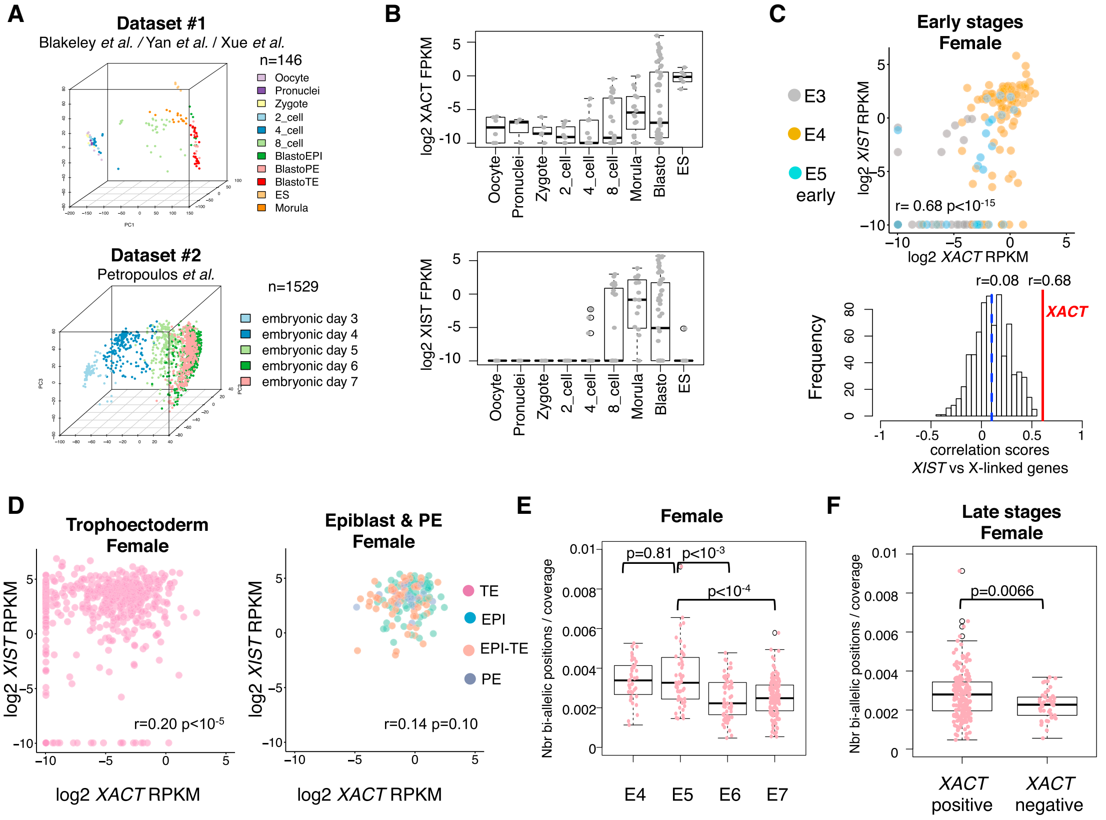

Figure 1. *XACT* and *XIST* Are Co-expressed in the Early Stages of Human Development

(A) Principal component analysis illustrates the developmental trajectory within the two datasets used in this study, based on the n = 1,000 most variant GENCODE genes within each dataset.

(B) Boxplot of *XACT* and *XIST* expression levels (log2 FPKM) according to developmental stage, from dataset 1.

(C) Upper panel: plot of *XACT* versus *XIST* expression levels (log2 reads per kilobase per million mapped reads [RPKM]) in early-stage female cells of dataset 2 (E3, E4, and early E5), with corresponding Spearman correlation score and p value. Lower panel: distribution of Spearman correlation scores between *XIST* and each X-linked gene expression level. The median correlation score is indicated as a blue dashed line and a red line indicates the correlation score with *XACT* expression levels.

(D) Plots of *XACT* versus *XIST* expression levels (log2 RPKM) in late-stage female cells of dataset 2 (E5, E6, and E7), in trophectoderm (left panel) and epiblast and primitive endoderm cells, as assessed by a 75-gene signature shown in Figure S1C. The corresponding Spearman correlation score and p value are indicated.

(E) Boxplot of the number of X-linked bi-allelic positions normalized by coverage according to day of development for dataset 2. The p values of a Wilcoxon rank test comparing distributions between stages are indicated above the boxplot.

(F) Boxplot of the number of X-linked bi-allelic positions normalized by coverage for *XACT*-positive and -negative cells in female late-stage blastocysts. The p value of a Wilcoxon rank test comparing both distributions is indicated above the boxplot.

of the inactive state are spontaneously and irreversibly lost in culture. This erosion of XCI is characterized by the loss of *XIST* expression, loss of H3K27me3 enrichment, DNA hypomethylation, and partial gene reactivation (Mekhoubad et al., 2012; Nazor et al., 2012; Vallot et al., 2015). Accumulation of *XACT* is also a feature of the eroded X and occurs early during the erosion process, prior to the loss of *XIST* and to gene reactivation (Vallot et al., 2015). This order of events suggests that *XACT* could participate in the instability of XCI in hESCs by influencing *XIST* expression, *XIST* RNA activity, or localization.

Here we show that *XACT* is expressed in human pre-implantation embryos, where it accumulates, together with *XIST*, on active X chromosomes. We also report an in vitro context of naive pluripotency that recapitulates the in vivo situation, thus defining a unique pre-inactivation state in human development. Functional evidences further indicate that *XACT* influences *XIST* accumulation in *cis*, suggesting that dosage compensation establishment in human involves the antagonistic action of two lncRNAs.

RESULTS

XIST and *XACT* Expression Profiling from Single-Cell RNA-Seq Human Pre-implantation Embryo Datasets

To investigate the biological relevance of *XACT* and its putative function in XCI, we probed its expression in vivo, in the early stages of human development. We analyzed four independent sets of single-cell RNA sequencing (RNA-seq) data generated from human pre-implantation embryos, which we grouped into two datasets (Figure 1A) based on the methods used to classify embryos. In dataset 1 (Blakeley et al., 2015; Xue et al., 2013; Yan et al., 2013), embryos (41 embryos; n = 146 cells) are classified

by developmental stages, ranging from oocyte to blastocyst, whereas in dataset 2 (Petropoulos et al., 2016), embryos (88 embryos; n = 1,529 cells) are classified according to the day of development, ranging from embryonic day E3 to E7.

The major burst of zygotic *XIST* and *XACT* expression occurred concomitantly between the four-cell and the eight-cell stages (Figure 1B). *XIST* was undetectable prior to these stages, while there was a modest maternal contribution for *XACT* (average fragments per kilobase per million mapped reads [FPKM] = 0.005 from oocyte to two-cell stage). The expression of *XIST* and *XACT* was strongly correlated at early stages of development (from E3 to early E5) in both female and male embryos, suggesting that the two noncoding genes, which are located 40 Mb apart on the X, might be co-regulated (Figure 1C; Figure S1B). This correlation was likely significant and not only due to ZGA, as it was not observed for any other X-linked genes (lower panel, Figure 1C). However, *XIST-XACT* correlation did not persist in later-stage embryos (from E5 to E7, Figure S1D). In females, *XIST* and *XACT* were maintained co-expressed in epiblast (EPI and EPI-TE) and primitive endoderm (PE) cells. In contrast, trophectoderm (TE) cells displayed variable *XACT* levels (Figure 1D) while maintaining stable *XIST* expression. Such a pattern suggests that, once initiated, the expression of *XIST* and *XACT* can be regulated independently, in agreement with the persistence of *XIST* expression from the inactive X chromosome in differentiated cells where *XACT* is repressed (Vallot et al., 2013).

We next exploited both datasets to follow the dynamics of *XACT* and *XIST* expression in the context of XCI. Applying a dedicated pipeline based on Genome Analysis Toolkit (GATK) tools (McKenna et al., 2010), we used the normalized number of X-linked bi-allelic positions as a marker of X chromosome activity in female cells (Figures S1E and S1F). The number of bi-allelic positions on the X decreased significantly with developmental stage in both datasets compared to several autosomes (Figure 1E; Figures S1G and S1H), indicative of a potential initiation of X chromosome silencing. Although this contrasts somewhat with a recent report that concluded an absence of XCI at these stages (Petropoulos et al., 2016), the discrepancy may be due to the choice of threshold for calling bi-allelic positions (i.e., allelic ratio, Figure S1I). Altogether, these findings suggest the existence of an expression imbalance between the two X chromosomes starting at E6, which can be interpreted as an initiation of XCI. Strikingly, *XACT*-negative cells were the ones displaying the lowest numbers of X-linked bi-allelic positions (Figure 1F, p = 0.0066), potentially linking in time the repression of *XACT* to the initiation of XCI.

XIST and *XACT* Co-accumulate on X Chromosomes in Pre-implantation Embryos

Single-cell RNA-seq analysis thus revealed that *XACT* and *XIST* can be simultaneously expressed within a given cell and likely from a given chromosome, as extrapolated from male samples. The known ability of these two noncoding transcripts to individually coat the chromosome from which they are expressed prompted us to investigate their nuclear distributions at the single-cell level in human pre-implantation embryos. This also enabled us to refine our understanding of the timing and allelism of expression and the localization of *XACT* and *XIST* by precise developmental scoring of embryos, through the use of a blastocyst morphological classification (Gardner et al., 2000) and the assessment of total cell counts for each embryo. RNA-fluorescence in situ hybridization (FISH) analysis of one female morula and eight male and 11 female blastocysts not only confirmed the expression of *XACT* at these developmental stages but also further demonstrated that *XACT* retains its ability to form an RNA cloud in vivo (Figure 2A). *XACT* coated the single X chromosome in males and one or both Xs in the majority of cells of female morula and early blastocysts, with a tendency to shift from bi- to mono-allelic expression as female blastocysts increased in cell number (Figure S2A). The overall proportion of cells with *XACT* expression decreased as development progressed (Figure 2B). Our prior RNA-seq analysis together with visual observation of labeled blastocysts suggests that *XACT*-negative cells are from the TE.

Simultaneous RNA-FISH for *XIST* and *XACT* in male and female blastocysts showed that both transcripts were often co-expressed from the X chromosome and accumulated in large domains within the nucleus (Figure 2C). *XIST* and *XACT* RNAs were, however, differentially distributed and the two signals barely overlapped (Figure 2C, zooms, Figure 2D), indicating that *XIST* and *XACT* transcripts occupy distinct nuclear territories. While the expression of *XIST* remained constant from the early (B1) to late/hatching (B5) blastocyst stage (Figure 2E), the proportion of cells with co-accumulation of *XACT* and *XIST* decreased according to the blastocyst cell number (Figure S2B), in agreement with the loss of *XACT* expression in a subset of blastocyst cells. In addition, no recurrent pattern could be identified when the relative allelic expression of *XIST* and *XACT* were compared (Figure S2C), confirming that, once activated, *XIST* and *XACT* can be regulated independently and suggesting some degree of stochasticity in their expression in blastocysts.

XIST and *XACT* Co-accumulate on Active X Chromosome in Naive Pluripotent Stem Cells

In vivo pre-inactivation status in human is thus characterized by the co-accumulation of *XACT* and *XIST* on active X chromosomes, in both males and females. hESCs in a pre-inactive state have been reported (Gafni et al., 2013; Lengner et al., 2010; Tomoda et al., 2012; Ware et al., 2014), but this status appears to be difficult to generate and maintain in culture. In addition, determining the X chromosome activity status in hESCs could prove challenging, given the spontaneous propensity of the inactive X to undergo partial reactivation in cultured hESCs and the resulting confusion between pre-inactive and eroded states (Vallot et al., 2015). More importantly, none of the naive-state hESCs reported so far have displayed *XIST* expression, as would be predicted from the data presented here and from previous analysis of *XIST* RNA status in human embryos (Okamoto et al., 2011). Additional protocols to capture naive hESCs have been described recently; in these cases, the epigenetic, metabolic, and transcriptomic signatures were strongly indicative of naive-state pluripotency (Takashima et al., 2014; Theunissen et al., 2014), but the status of the X chromosome had not been investigated in depth.

We first studied in primed H9 female hESCs and in their naive derivatives (Takashima et al., 2014) the expression of *ATRX* and *FGD1*, since these two X-linked genes resist XCI erosion

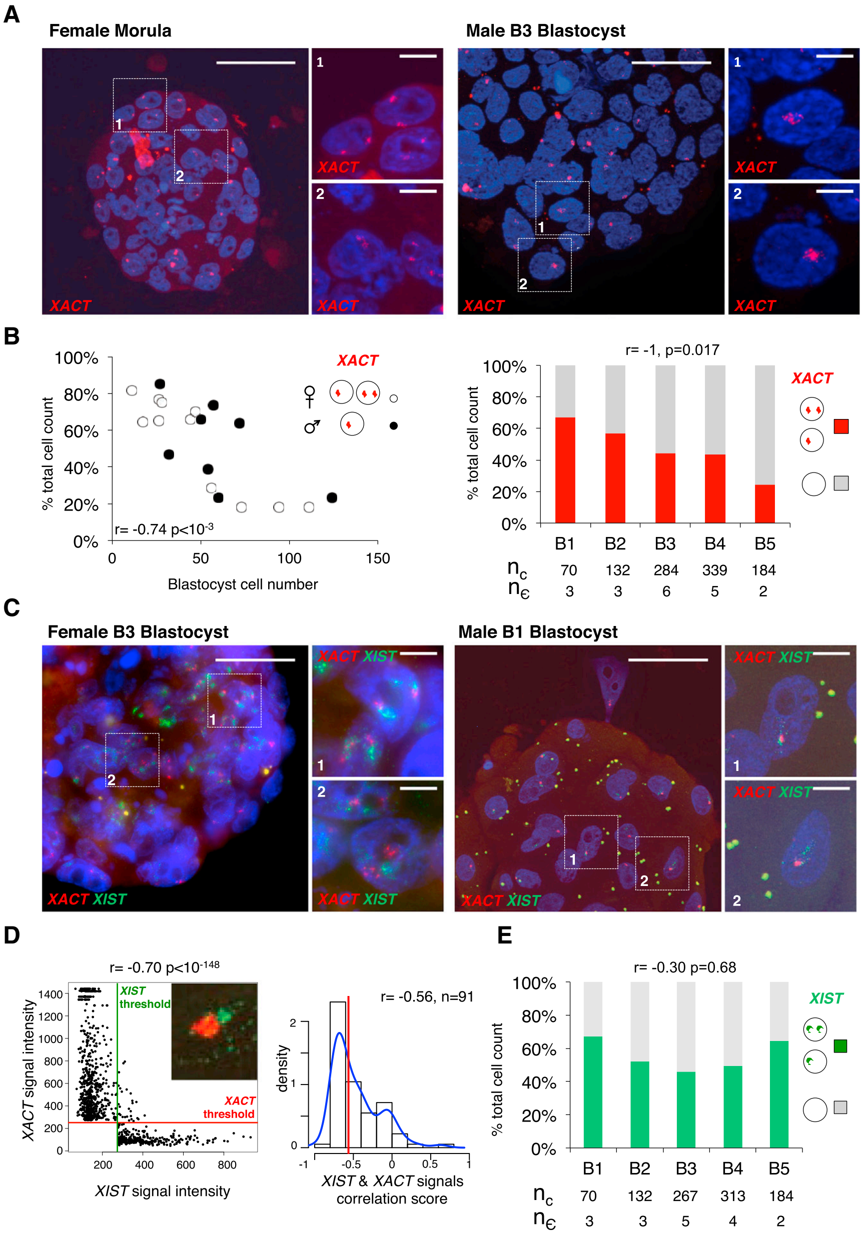

Figure 2. *XACT* Co-accumulates with *XIST* on the X Chromosome(s) in Human Pre-implantation Embryos

(A) Examples of female and male embryos analyzed by RNA-FISH to detect *XACT* expression.

(B) Left panel: the proportion of cells per male (dark circles) and female (white circles) blastocyst displaying *XACT* expression was plotted against blastocyst cell number. Right panel: the percentage of cells with *XACT* expression at each blastocyst stage (right), from B1 to B5, is shown. Below the graphs are indicated total cell number (n_c) and the number of embryos (n_ε) analyzed. The Spearman correlation score between the proportion of *XACT*-positive cells and the developmental stage is indicated.

(C) Simultaneous analysis of *XIST* (in green) and *XACT* (in red) expression by RNA-FISH in female and male blastocysts. White scale bars represent 50 μm for total embryos and 5 μm for zooms.

(D) Left panel: scatterplot representing the intensity of *XIST* versus *XACT* signal above respective thresholds for one nucleus. The red horizontal line and the green vertical line represent computed thresholds for *XACT*- and *XIST*-associated images, respectively. Signals were compared using a Spearman correlation test. Right panel: distribution of Spearman correlation scores for n = 91 nuclei is shown. A red line indicates the median correlation score and a blue line displays the density plot.

(E) Percentage of cells with *XIST* accumulation at each blastocyst stage, from B1 to B5. Below the graphs are indicated total cell number (n_c) and the number of embryos (n_ε) analyzed. The Spearman correlation score between the proportion of *XIST*-positive cells and the developmental stage is indicated.

(Vallot et al., 2015) yet are expressed from the two Xs in female pre-implantation embryos (Okamoto et al., 2011). Both genes were indeed mono-allelically expressed in the parental, primed hESCs. In striking contrast, two pinpoints for *ATRX* and *FGD1* were detected in 84% and 83%, respectively, of the naive cells, demonstrating that the conversion from primed to naive states was accompanied by substantial X chromosome reactivation (XCR) (Figures 3A and 3B). The presence of two active Xs in naive cells was further confirmed by allelic analysis of RNA-seq datasets (GEO: GSE60945 of Takashima et al., 2014), which revealed an increase in bi-allelically expressed genes in the naive state as compared to primed cells (from 55% to 76%, p = 0.0070, Figure 3C). More strikingly, while *XIST* was fully and uniformly repressed in the parental primed cells, indicating that these cells had undergone XCI erosion, *XIST* was strongly upregulated in naive hESCs, and it accumulated in the nucleus in over 80% of naive H9 on one or two Xs (Figures 3A and 3B). The expression of *XIST* was, however, highly unstable, and the percentage of cells with two or even one *XIST* RNA cloud displayed high batch-to-batch variability. This variation occurred independently of the activity status of the X chromosome, which remained remarkably constant (Figure 3D). Bi-allelic *ATRX* expression and *XIST* accumulation on one or both Xs was similarly observed in an independent naive cell line WIBR3 (Figure 3E) (Theunissen et al., 2014). We also investigated whether the commercially available RSeT-defined medium (STEMCELL Technologies) could efficiently revert XCI, but we did not detect bi-allelic expression of *ATRX* or *XIST* expression, revealing that XCR did not occur in these conditions (Figures S3A–S3D).

We probed the chromatin landscape of the X chromosomes in naive female H9 hESCs. Confocal analyses of immunofluorescence (IF) profiles for various histone marks combined to *XIST* RNA-FISH first showed an absence of H3K27me3 and H3K9me3 within the *XIST* RNA domain (Figure 3F), indicating that *XIST* accumulation in this context was not sufficient to

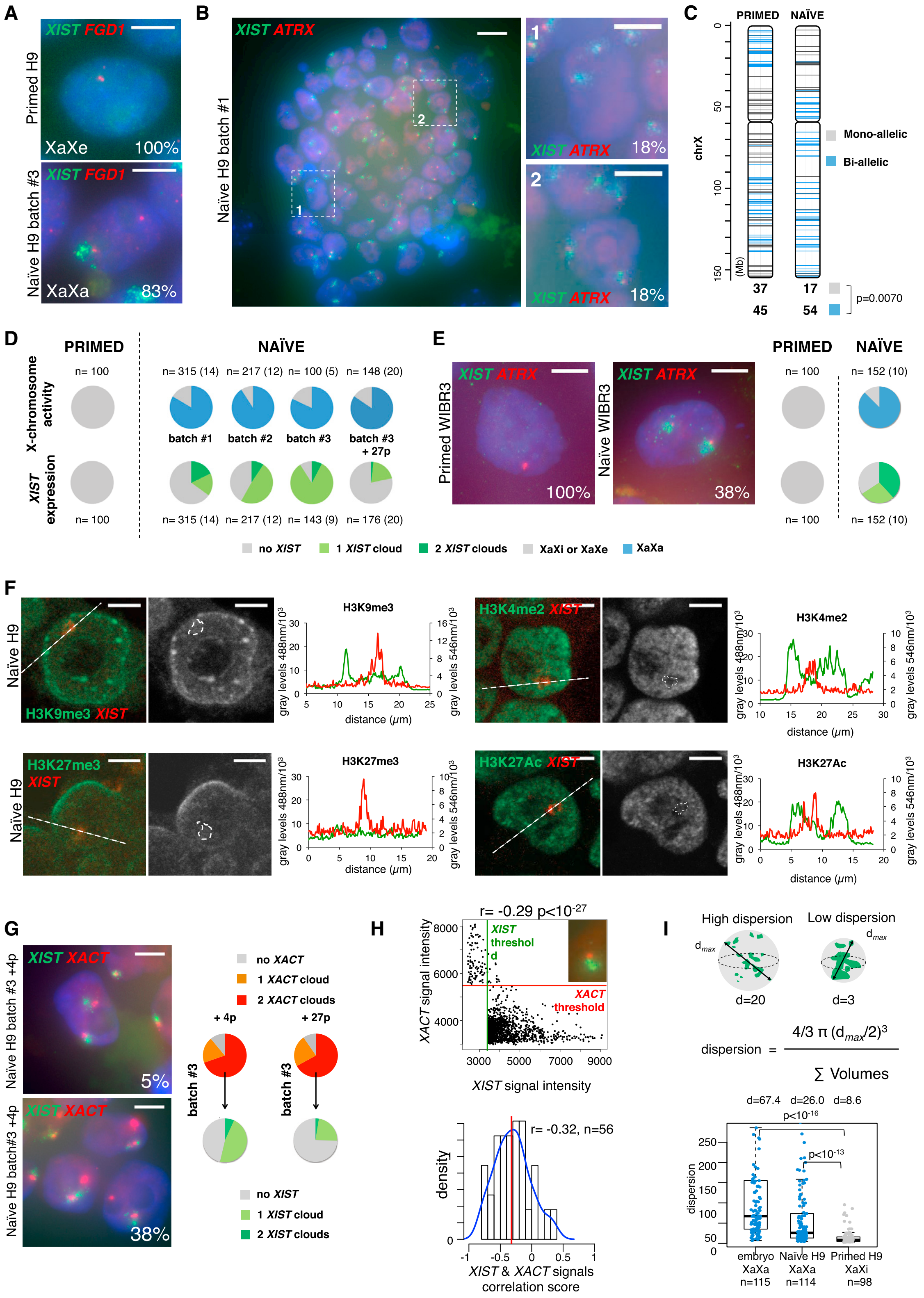

Figure 3. Naive hESCs Carry Active X Chromosomes Coated by *XIST*

(A) Analysis of *XIST* (in green) and *FGD1* (in red) expression by RNA-FISH in female H9 primed hESCs and their naive derivatives. The numbers indicate the percentage of cells with the displayed expression pattern. Primed cells have one active X chromosome (Xa) and one X chromosome that has undergone erosion of XCI (Xe). Naive cells carry two active Xs (XaXa). The white scale bars represent 5 µm.

(legend continued on next page)

trigger recruitment of repressive histone marks. This was reminiscent of the situation in human embryos where no H3K27me3 enrichment was detected on the *XIST*-coated chromosome (Okamoto et al., 2011). Analysis of published chromatin immunoprecipitation sequencing (ChIP-seq) datasets (Theunissen et al., 2014, 2016) confirmed the lack of H3K27me3 and H3K9me3 enrichment on the X in naive hESCs compared to the Xi in *XIST*-expressing primed cells (Figure S3E). Strikingly, IF analysis of H3K4me2 and H3K27Ac further revealed that the X chromosomes in naive hESCs, although active, resided in nuclear territories relatively devoid of active histone marks (Figure 3F).

We next investigated *XACT* expression in naive hESCs by RNA-FISH. *XACT* was expressed and accumulated on every X chromosome in the majority of naive H9 and WIBR3 cells (Figure 3G; Figure S3F), and this pattern was stably maintained in culture (Figure 3G). In cells in which *XIST* also was expressed, the two lncRNAs, although produced from the same chromosome(s), accumulated in distinct nuclear domains; as in embryos, no overlap could be detected between *XIST* and *XACT* signals (Figure 3H, median Spearman correlation score $r = -0.32$). Reinforcing the similarity between naive hESC and pre-implantation embryos, qualitative analysis revealed a much more dispersed *XIST* RNA-FISH signal in conditions where the Xs were active (embryos and naive hESCs) than in *XIST*-expressing primed hESCs (Wilcoxon rank test p value $< 10^{-16}$ and $p < 10^{-13}$, respectively), in which *XIST* coated an inactive X chromosome (Figure 3I). The different distribution of the *XIST* signal in naive versus primed pluripotent cells might underlie its limited ability to silence the chromosome in *cis*.

XACT Expression Influences *XIST* Accumulation in a Transgenic Context

The dispersed nature of the *XIST* RNA signal on active X chromosomes in human embryos and naive hESCs where it accumulates with *XACT*, together with the lack of co-localization of *XIST* and *XACT*, raises the hypothesis that *XACT* might perturb the tight localization of *XIST* across the chromosome and/or its silencing capacities. To test these hypotheses, we sought to generate a system in which *XACT* expression would precede *XIST* upregulation. For this, we inserted a BAC transgene containing a large part of *XACT* including its promoter region into mouse ESCs. By using first an untargeted approach, we obtained one clone (clone R1) in which insertion of the *XACT* BAC randomly occurred on one of the two Xs, distal to the *Xist* locus, as shown by DNA-FISH on metaphase spreads (Figure 4B). We next used the CRISPR/Cas9 technology to force the insertion of the BAC onto the X, by targeting the integration between the protein-coding genes *Amot* and *Htr2c*, which corresponds to the *XACT* syntenic region on the mouse X chromosome (Figure 4A) (Vallot et al., 2013). Using this strategy, we isolated several clones with transgene insertion on the X, two of which were selected for further investigation (T1 and T9). As the control we used clones in which an unrelated BAC containing the human *FGD1* gene was similarly inserted on the X and clones in which *XACT* was inserted on autosomes (Figure S4A).

XACT expression was specifically detected in the clones with *XACT* BAC transgenes, and in most clones *XACT* formed an RNA cloud in a large proportion of cells (Figure 4B; Figure S4A). The copy number of inserted BACs for each of these clones was determined by real-time qPCR (Figure S4E). *Xist* was appropriately upregulated, albeit to variable extents, when transgenic clones were induced to differentiate, while *XACT* expression tended to decrease upon differentiation, similar to what has been observed in humans (Vallot et al., 2013) (Figures S4B and S4C). Within the population of cells in which *XACT* expression was maintained, we observed a significant bias in the choice of the *Xist*-coated chromosome, with *Xist* being preferentially upregulated from the X not carrying *XACT* in the R1 and T9 clones (Figure 4C; Fisher's exact test, $p < 10^{-4}$). However, in the T1 clone (as in clones in which the *FGD1* BAC was integrated on the X, Figure S4D), *Xist* was able to accumulate similarly on the transgenic and wild-type (WT) X chromosomes (Figure 4C; Figure S4F). Variation in the expression levels of *XACT* is unlikely

(B) Analysis of *XIST* (in green) and *ATRX* (in red) expression by RNA-FISH in naive H9 cells. Panels 1 and 2 are zooms taken within the colony displayed on the left. The white scale bars represent 10 μm for the image of the colony (left panel) and 5 μm for the zooms (right panels).

(C) Allelic analysis of RNA-seq data generated from H9 primed (left) and naive (right) cells (Takashima et al., 2014). Informative positions gathered from the H9 genome (Vallot et al., 2015) showing mono-allelic expression are indicated in gray, those with bi-allelic expression in blue. Numbers of mono-allelic and bi-allelic genes in primed and naive cells are indicated beneath each cartoon. Numbers of bi- and mono-allelically expressed genes were compared using a Fisher's exact test.

(D) Upper panel: quantification of XaXa (blue) and XaXi/XaXe (gray) cells in primed and in several batches of naive H9 cells. The XaXa status was defined based on bi-allelic expression of *FGD1*. n, the number of cells counted; in brackets are indicated the number of colonies. Lower panel: similar quantification of cells with zero (gray), one (light green), and two (dark green) *XIST* RNA clouds is shown. p, passage number.

(E) Assessment of *ATRX* (in red) and *XIST* (in green) expression in primed and naive female WIBR3 cells. Quantification is as in (D), except that the XaXa status in that case was defined based on bi-allelic expression of *ATRX*.

(F) Confocal analysis of immunofluorescence (IF) for H3K9me3, H3K27me3, H3K4me2, and H3K27Ac (in green) coupled to *XIST* RNA-FISH (in red). A representative image is shown for each histone mark, and panels on the right display quantification of gray levels for IF and RNA-FISH signals along the white dashed lines within a single z section. The white scale bar represents 5 μm.

(G) Assessment of *XIST* (in green) and *XACT* (in red) expression by RNA-FISH and quantification of the observed profiles in two different batches of naive H9 cells. p, passage number.

(H) Left panel: scatterplot representing the intensity of *XIST* versus *XACT* signal above respective thresholds for one nucleus. The red horizontal line and the green vertical line represent computed thresholds for *XACT*- and *XIST*-associated images, respectively. Signals were compared using a Spearman correlation test. Right panel: distribution of Spearman correlation scores for n = 56 nuclei. A red line indicates the median correlation score and a blue line displays the density plot.

(I) Left panel: model for the computation of a dispersion value for each *XIST* signal. Right panel: boxplot illustrates the distribution of the dispersion of *XIST* RNA-FISH signal in embryos and in primed and naive H9 and WIBR2 cells. Median dispersions are indicated above the boxplot for each group, Wilcoxon rank test was used to compare distributions of *XIST* signal dispersion among primed H9 cells, naive H9 cells, and embryo cells.

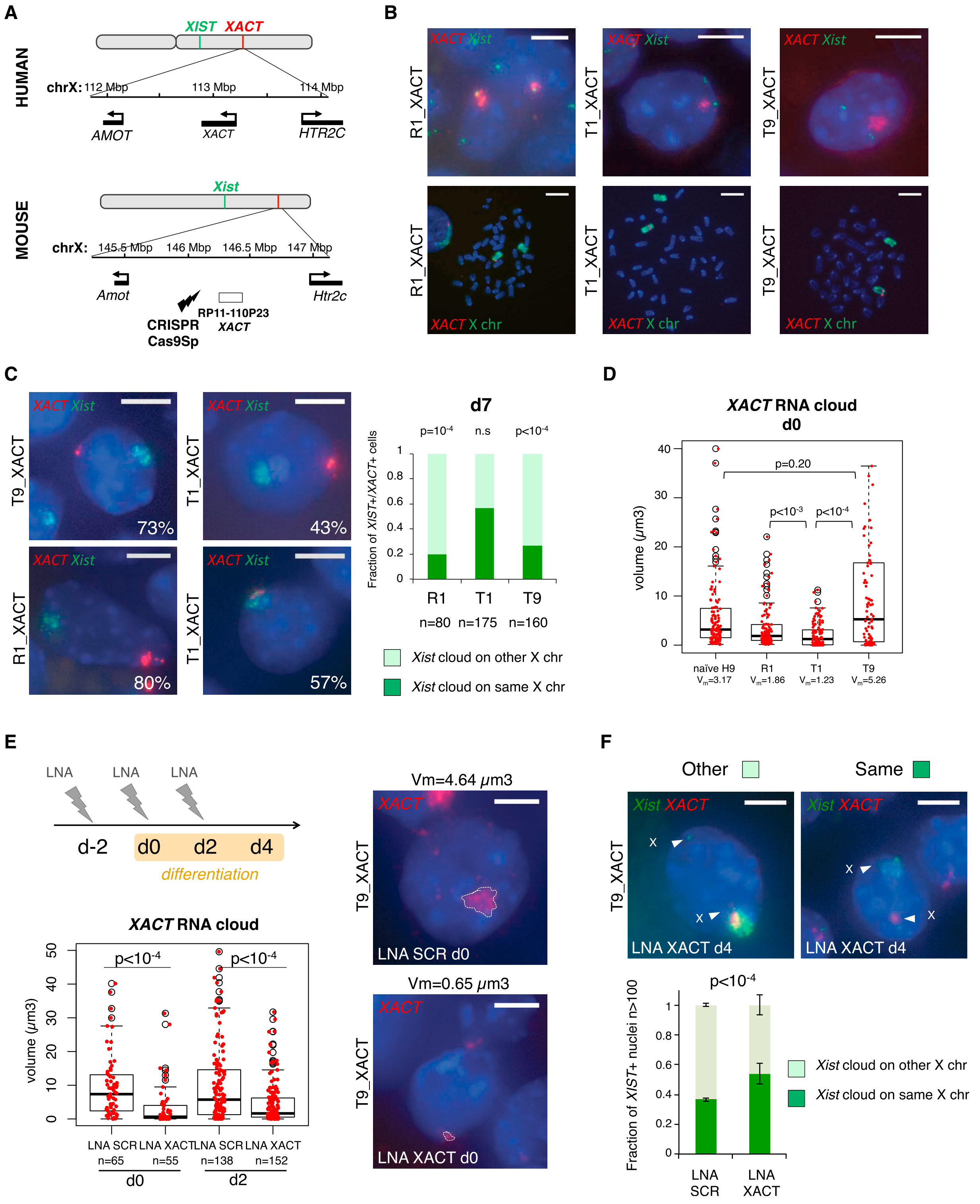

Figure 4. Inserting *XACT* on a Mouse X Chromosome Influences the Choice of the Inactive X

(A) Map of the *XACT* locus in human and of the syntenic region on the mouse X chromosome into which the *XACT* BAC was integrated.

(B) Assessments of *XACT* and *Xist* expression in interphase nuclei of undifferentiated mouse ESCs (upper panels) and of their localization on metaphase chromosomes (lower panels) in clones with random (R1) or targeted (T1 and T9) integration of *XACT*.

(C) Assessment of the chromosome of origin for *Xist* expression related to *XACT* integration in *Xist*- and *XACT*-positive cells at day 7 of differentiation, using RNA-FISH for *Xist* (in green) and *XACT* (in red). Barplot on the right panel shows the quantification of the respective expression patterns in one representative experiment, which were compared using a Fisher's exact test.

(legend continued on next page)

to account for those differences, as the T1 clone expressed intermediate levels of *XACT* compared to R1 and T9 clones (Figure S4B). However, we found that the nuclear volume occupied by *XACT* was correlated with its ability to bias *Xist* expression (Figure 4D). *XACT* nuclear volume in R1 and T9 clones was in the same range as in naive hESCs and significantly higher than in the T1 clone. The comparison between the T1 and T9 clones is particularly relevant as they shared the same insertion site for *XACT*.

To further investigate the impact of *XACT* RNA on *Xist* accumulation, we knocked down *XACT* in the T9 clone using LNA Gapmers. Successive rounds of knockdown (KD) both prior to and at the onset of differentiation induced a strong decrease in *XACT* RNA levels and in the *XACT* RNA cloud volume (Figure 4E; Figure S4G). By performing simultaneous RNA-DNA-FISH experiments to assess the localization of the *Xist* cloud with respect to the *XACT* transgene (independently of *XACT* expression), we showed that *XACT* KD reverted XCI to random, with *Xist* accumulating in the same proportion on the WT or on the *XACT* transgenic X chromosome (Figure 4F). Altogether, our data demonstrate that robust accumulation of *XACT* on one X chromosome directly impacts the expression or localization of *Xist* in *cis*.

DISCUSSION

Here, by benchmarking the naive state of pluripotency to the human embryo, we have demonstrated that the pre-XCI state in humans is characterized by the simultaneous accumulation of *XIST* and *XACT* on active X chromosomes. This scenario, which differs radically from the mouse, highlights the plasticity of epigenetic regulations across species and the contribution of lncRNAs to species specificity.

During human development, *XIST* expression and accumulation on X chromosomes begin rapidly after ZGA, yet it takes several divisions before *XIST* initiates XCI. While we confirm here the lack of chromosome-wide XCI prior to implantation, our allelic analysis of RNA-seq data points to an initiation of silencing at the late pre-implantation stages, which would act initially at a gene-to-gene level. We moreover show that active X chromosomes decorated by *XIST* are also characteristic of naive-state pluripotency in vitro. *XIST* expression is, however, highly unstable in this context, suggesting that culture conditions to maintain cells in the naive state are not yet optimum and that *XIST*-positive cells might be counter-selected in current conditions. Bi-allelic *XIST* accumulation might thus be used as a biomarker for defining naive human pluripotency, in the search for refined growth conditions. In addition, during the transition from primed to naive pluripotency, XCR appears to be uncoupled from the major transcriptional and morphological resetting of the cells and likely occurs late in the course of the reprogramming process.

Our findings reveal that *XIST* adopts a peculiar, more dispersed configuration in naive contexts, which may underlie its poor silencing ability. In addition, this scattered accumulation appears not sufficient to recruit repressive histone marks on the X chromosomes. We furthermore show that pre-inactive X chromosomes also accumulate *XACT*, both in vitro and in vivo, demonstrating that *XACT* expression is not restricted to cultured cells and further highlighting its biological relevance. The concomitant activation of *XIST* and *XACT* following fertilization is suggestive of a concerted action in a common biological process.

The facts that *XIST* and *XACT* RNAs co-accumulate yet barely overlap and that introducing *XACT* into a heterologous system influences *Xist* RNA accumulation in *cis* further suggest that *XACT* may act by controlling the association of *XIST* to the chromosome in *cis*, possibly to antagonize or temper its silencing ability. Alternatively, *XACT* could directly participate in the compensatory mechanism occurring at these early stages (Petropoulos et al., 2016), by controlling X chromosome transcriptional outcome. In both cases, this raises the hypothesis that *XACT* function might be linked to the lack of tight control of *XIST* expression in early human development. In this scenario, *XACT* might have evolved to prevent X chromosome silencing and functional nullisomy and to permit an alternative strategy of dosage compensation at these critical developmental stages.

EXPERIMENTAL PROCEDURES

Comprehensive Analysis of Single-Cell RNA-Seq Datasets

We gathered single-cell RNA-seq samples from four independent studies on human embryos (Blakeley et al., 2015; Petropoulos et al., 2016; Xue et al., 2013; Yan et al., 2013). We merged together the samples from the three studies (dataset 1 [Yan et al., 2013; Xue et al., 2013; Blakeley et al., 2015], n = 146) where embryos had been classified according their developmental stage, whereas in the last study (dataset 2 [Petropoulos et al., 2016], n = 1,529) embryos were classified according to their last day of in vitro culture.

Assessment of XCI in RNA-Seq Samples

We used the normalized number of bi-allelic positions on the X chromosome in the RNA-seq datasets as a marker of the activity of the two X chromosomes in female cells. A different number of bi-allelic positions between two conditions is indicative of a change in the transcription balance of two X chromosomes. We also repeated the analysis performed by Petropoulos et al. (2016) to compare with our results.

(D) Boxplot representing the distribution of the volume of the *XACT* RNA-FISH signal in naive H9 and in transgenic mESC clones. Wilcoxon rank test was used to compare the volume of *XACT* signals.

(E) LNA Gapmer-mediated *XACT* KD. Upper panel: timeline indicating the KD strategy during differentiation of the T9 transgenic clone is shown. Three rounds of LNA transfection were performed: one 2 days before the induction of differentiation (d-2), one concomitantly with the launching of differentiation (d0), and the last at day 2 (d2) of differentiation. Lower panel: boxplot representing the distribution of the volume of the *XACT* RNA-FISH signal in cells transfected with either scramble or *XACT* LNA, both at d0 and d2 of differentiation, is shown. Wilcoxon rank test was used to compare the volume of *XACT* signals between KD and control cells. Representative *XACT* RNA-FISH images corresponding the median volume for LNA scramble and LNA *XACT* are shown, with the *XACT* volume delimited by the dotted white line.

(F) Assessment of the chromosome of origin for *Xist* expression related to *XACT* integration at day 4 of differentiation of cells transfected with either scramble or *XACT* LNA, using RNA-FISH for *Xist* (in green) and DNA-FISH for *XACT* (in red). Barplot on the right panel shows the quantification of the respective expression patterns for three independent KD experiments, the error bars corresponding to the SD. Patterns were compared independently for each experiment using a Fisher's exact test; the p value was always below 10^{-4}. The white scale bars in RNA-FISH images represent 5 μm and 10 μm for metaphases.

Collection of Human Embryos and RNA-FISH

French Biomedecine Agency authorization was obtained for the experimental use of supernumerary cryopreserved embryos resulting from infertility treatment and donated for research (RE 10-032R/RE 12-012R). Written consents were obtained from the couples that their cryopreserved embryos could be used for the research. Human cryopreserved embryos were obtained at Bichat Hospital (Assistance Publique – Hôpitaux de Paris) after in vitro fertilization (IVF) and intra-cytoplasmic sperm injection (ICSI). Day 2–3 cryopreserved embryos were thawed (Embryo Thawing Pack, Origio) and were individually placed in fresh and equilibrated 30 μl culture medium (ISM1 culture medium from day 2 to day 3 and Blastassist from day 3 and on, Origio) at 37°C, 5% CO_2, under oil and humidified atmosphere. Embryos were cultivated until day 4 (morula) or day 5 and 6 (blastocyst stage). Blastocysts were evaluated using a Leica AM 6000 B inversed microscope at ×400 by an experienced embryologist. An embryo was included in this study only if the embryologist agreed on viability. Blastocysts were classified according to Gardner's classification (Gardner et al., 2000), taking into account the global morphology and inner cell mass and TE aspect from mid-blastocyst. RNA-FISH was carried out as described previously (Okamoto et al., 2011) (see the Supplemental Experimental Procedures).

SUPPLEMENTAL INFORMATION

Supplemental Information includes Supplemental Experimental Procedures and four figures and can be found with this article online at http://dx.doi.org/10.1016/j.stem.2016.10.014.

AUTHOR CONTRIBUTIONS

C.V. and C.R. conceived the project and planned experiments. C.V., C.H., M.C., and M.T. performed the experiments. C.P., N.F., and E.H. provided and contributed to the analysis of human embryos. A.J.C. and P.J.R.-G. provided and performed experiments on naive hESCs. T.M.L.A. and C.V. performed bioinformatic analyses. C.V. and C.R. wrote the manuscript. C.P., A.J.C., and C.H. contributed equally to this work. All authors commented on and revised the manuscript.

ACKNOWLEDGMENTS

We thank S. Polo and S. Ait Si Ali for critical reading of the manuscript. We thank P. Tuffery, J. Bécot, the ''Initiatives d'excellence'' Université Sorbonne Paris Cité (USPC) (ANR-11-IDEX-0005-02), and the LabEx ''Who Am I'' (ANR-11-LABX-0071) for computational support. We thank S. Abelanet at the ImagoSeine Institut Jacques Monod Imaging platform (Université Paris 7) for valuable advice and technical assistance on the use of the SP5 Leica microscope. We also thank L. Delaroche, J.-P. Wolf, M.-A. Llabador-de Royer, M. Lemoine, and A. Benammar for support on human embryo experiments. The research leading to these results has received funding from the European Research Council under the EpiGeneSys FP7 257082 Network of Excellence (to C.R., E.H., and P.J.R.-G.), from the Agence Nationale pour la Recherche (ANR-11-LABX-0071, to C.R. and E.H.), from the Ligue Nationale contre le Cancer (to C.R.), and from the DIM Biotherapies (to E.H. and C.P.). T.M.L.A. is supported by a fellowship from the INCa/INSERM Plan cancer (EPIG201414). P.J.R.-G. is supported by the Wellcome Trust (WT093736) and the Biotechnology and Biological Sciences Research Council (BBSRC) (BBS/E/B/000C0402).

Received: July 1, 2016
Revised: September 23, 2016
Accepted: October 19, 2016
Published: December 15, 2016

REFERENCES

Blakeley, P., Fogarty, N.M., del Valle, I., Wamaitha, S.E., Hu, T.X., Elder, K., Snell, P., Christie, L., Robson, P., and Niakan, K.K. (2015). Defining the three cell lineages of the human blastocyst by single-cell RNA-seq. Development *142*, 3151–3165.

Chu, C., Zhang, Q.C., da Rocha, S.T., Flynn, R.A., Bharadwaj, M., Calabrese, J.M., Magnuson, T., Heard, E., and Chang, H.Y. (2015). Systematic discovery of Xist RNA binding proteins. Cell *161*, 404–416.

Disteche, C.M. (2016). Dosage compensation of the sex chromosomes and autosomes. Semin. Cell Dev. Biol. *56*, 9–18.

Gafni, O., Weinberger, L., Mansour, A.A., Manor, Y.S., Chomsky, E., Ben-Yosef, D., Kalma, Y., Viukov, S., Maza, I., Zviran, A., et al. (2013). Derivation of novel human ground state naive pluripotent stem cells. Nature *504*, 282–286.

Gardner, D.K., Lane, M., Stevens, J., Schlenker, T., and Schoolcraft, W.B. (2000). Blastocyst score affects implantation and pregnancy outcome: towards a single blastocyst transfer. Fertil. Steril. *73*, 1155–1158.

Lengner, C.J., Gimelbrant, A.A., Erwin, J.A., Cheng, A.W., Guenther, M.G., Welstead, G.G., Alagappan, R., Frampton, G.M., Xu, P., Muffat, J., et al. (2010). Derivation of pre-X inactivation human embryonic stem cells under physiological oxygen concentrations. Cell *141*, 872–883.

McHugh, C.A., Chen, C.K., Chow, A., Surka, C.F., Tran, C., McDonel, P., Pandya-Jones, A., Blanco, M., Burghard, C., Moradian, A., et al. (2015). The Xist lncRNA interacts directly with SHARP to silence transcription through HDAC3. Nature *521*, 232–236.

McKenna, A., Hanna, M., Banks, E., Sivachenko, A., Cibulskis, K., Kernytsky, A., Garimella, K., Altshuler, D., Gabriel, S., Daly, M., and DePristo, M.A. (2010). The Genome Analysis Toolkit: a MapReduce framework for analyzing next-generation DNA sequencing data. Genome Res. *20*, 1297–1303.

Mekhoubad, S., Bock, C., de Boer, A.S., Kiskinis, E., Meissner, A., and Eggan, K. (2012). Erosion of dosage compensation impacts human iPSC disease modeling. Cell Stem Cell *10*, 595–609.

Minajigi, A., Froberg, J.E., Wei, C., Sunwoo, H., Kesner, B., Colognori, D., Lessing, D., Payer, B., Boukhali, M., Haas, W., and Lee, J.T. (2015). Chromosomes. A comprehensive Xist interactome reveals cohesin repulsion and an RNA-directed chromosome conformation. Science *349*, aab2276.

Nazor, K.L., Altun, G., Lynch, C., Tran, H., Harness, J.V., Slavin, I., Garitaonandia, I., Müller, F.J., Wang, Y.C., Boscolo, F.S., et al. (2012). Recurrent variations in DNA methylation in human pluripotent stem cells and their differentiated derivatives. Cell Stem Cell *10*, 620–634.

Okamoto, I., Patrat, C., Thépot, D., Peynot, N., Fauque, P., Daniel, N., Diabangouaya, P., Wolf, J.P., Renard, J.P., Duranthon, V., and Heard, E. (2011). Eutherian mammals use diverse strategies to initiate X-chromosome inactivation during development. Nature *472*, 370–374.

Petropoulos, S., Edsgärd, D., Reinius, B., Deng, Q., Panula, S.P., Codeluppi, S., Plaza Reyes, A., Linnarsson, S., Sandberg, R., and Lanner, F. (2016). Single-cell RNA-seq reveals lineage and X chromosome dynamics in human preimplantation embryos. Cell *165*, 1012–1026.

Takashima, Y., Guo, G., Loos, R., Nichols, J., Ficz, G., Krueger, F., Oxley, D., Santos, F., Clarke, J., Mansfield, W., et al. (2014). Resetting transcription factor control circuitry toward ground-state pluripotency in human. Cell *158*, 1254–1269.

Theunissen, T.W., Powell, B.E., Wang, H., Mitalipova, M., Faddah, D.A., Reddy, J., Fan, Z.P., Maetzel, D., Ganz, K., Shi, L., et al. (2014). Systematic identification of culture conditions for induction and maintenance of naive human pluripotency. Cell Stem Cell *15*, 471–487.

Theunissen, T.W., Friedli, M., He, Y., Planet, E., O'Neil, R.C., Markoulaki, S., Pontis, J., Wang, H., Iouranova, A., Imbeault, M., et al. (2016). Molecular criteria for defining the naive human pluripotent state. Cell Stem Cell *19*, 502–515.

Tomoda, K., Takahashi, K., Leung, K., Okada, A., Narita, M., Yamada, N.A., Eilertson, K.E., Tsang, P., Baba, S., White, M.P., et al. (2012). Derivation conditions impact X-inactivation status in female human induced pluripotent stem cells. Cell Stem Cell *11*, 91–99.

Vallot, C., Huret, C., Lesecque, Y., Resch, A., Oudrhiri, N., Bennaceur-Griscelli, A., Duret, L., and Rougeulle, C. (2013). XACT, a long noncoding transcript coating the active X chromosome in human pluripotent cells. Nat. Genet. *45*, 239–241.

Vallot, C., Ouimette, J.F., Makhlouf, M., Féraud, O., Pontis, J., Côme, J., Martinat, C., Bennaceur-Griscelli, A., Lalande, M., and Rougeulle, C. (2015).

Erosion of X chromosome inactivation in human pluripotent cells initiates with XACT coating and depends on a specific heterochromatin landscape. Cell Stem Cell *16*, 533–546.

Ware, C.B., Nelson, A.M., Mecham, B., Hesson, J., Zhou, W., Jonlin, E.C., Jimenez-Caliani, A.J., Deng, X., Cavanaugh, C., Cook, S., et al. (2014). Derivation of naive human embryonic stem cells. Proc. Natl. Acad. Sci. USA *111*, 4484–4489.

Xue, Z., Huang, K., Cai, C., Cai, L., Jiang, C.Y., Feng, Y., Liu, Z., Zeng, Q., Cheng, L., Sun, Y.E., et al. (2013). Genetic programs in human and mouse early embryos revealed by single-cell RNA sequencing. Nature *500*, 593–597.

Yan, L., Yang, M., Guo, H., Yang, L., Wu, J., Li, R., Liu, P., Lian, Y., Zheng, X., Yan, J., et al. (2013). Single-cell RNA-Seq profiling of human preimplantation embryos and embryonic stem cells. Nat. Struct. Mol. Biol. *20*, 1131–1139.

Cell Stem Cell, Volume *20*

Supplemental Information

XACT Noncoding RNA Competes with *XIST* in the Control of X Chromosome Activity during Human Early Development

Céline Vallot, Catherine Patrat, Amanda J. Collier, Christophe Huret, Miguel Casanova, Tharvesh M. Liyakat Ali, Matteo Tosolini, Nelly Frydman, Edith Heard, Peter J. Rugg-Gunn, and Claire Rougeulle

Supplementary Experimental Procedures

Comprehensive analysis of single-cell RNA-seq datasets

We gathered single-cell RNA-seq samples from four independent studies on human embryos. We gathered single-cell RNA-seq samples from four independent studies on human embryos: GSE36552 (Yan et al., 2013), GSE44183 (Xue et al., 2013), GSE66507 (Blakeley et al., 2015), E-MTAB-3929 (Petropoulos et al., 2016). All reads were aligned to the human genome (hg19) using Tophat2 (Kim et al., 2013) and only uniquely mapped reads were kept. Gene expression levels (Ensembl annotation GRCh37) were estimated as reads (or fragments for paired-end libraries) per kilobase of exon model and per million of mapped reads: RPKM (or FPKM). We first used htseq-count (Anders et al., 2015) to estimate the number of reads mapping to each gene within the reference annotation. Reads overlapping two genes were discarded. We then used an in-house script (R, https://www.r-project.org) to normalize the count matrices by coding length of each gene and by library size. Throughout the paper we used log2(RPKM +0.001) as expression levels for representation, unsupervised analysis and calculus of Spearman's correlation scores. All subsequent analysis were conducted in R. Principal Component analysis for the n=1000 most variant genes of each dataset (genes with the highest standard deviation) was used to represent the two datasets in Fig. 1a. To determine the sex of each cell within datasets #1 and #2 (and subsequently each embryo), we used the sum of the expression of the Y-linked genes (as in Petropoulos et al. (Petropoulos et al., 2016)). Seeing the bimodal distribution of the $\Sigma RPKM_{Ygenes}$ (Supplementary Fig. 1a), cells with $\Sigma RPKM_{Ygenes} < 50$ were classified as female and cells with $\Sigma RPKM_{Ygenes} > 100$ were classified as male. Since the zygotic genome activation occurs between the 4 and 8-cell stage, we could only determine with confidence the sex of cells from a stage later than 4-cell in dataset #1 and starting from E4 in dataset #2.

We used standard R functions to perform hierarchical clustering (with Euclidian distance and average linkage). We used consensus clustering (Bioconductor *ConsensusClusterPlus* package) (Wilkerson and Hayes, 2010) to examine the stability of the clusters. We established consensus partitions of the data set in K clusters (for K = 2, 3 to 10), on the basis of 1 000 resampling iterations (80% of genes, 80% of sample) of hierarchical clustering, with Euclidian distance as the distance metric and average method for linkage analysis. Using the cumulative distribution functions (CDFs) of the consensus matrices and its coupled plot of the relative change in area under the CDF curve (Supplementary Fig. 1c), we observed that a standard partition in k=3 clusters (TE-like, PE-like and EPI-like) was highly unstable, and samples often changed groups when performing repetitive clustering. We chose a partition in k= 4 clusters, named TE, PE, EPI and EPI-TE according to the pattern of expression of the 75-gene signature. We estimated the distribution and variability of *XACT* and *XIST* expression levels in female late stage blastocyst according to lineage, using the median value of log2 expression levels for each lncRNA in TE, PE, EPI and EPI-TE and its associated median absolute deviation (MAD).

Assessment of X-chromosome inactivation in RNA-seq samples

We started from the set of uniquely aligned reads (see above). We filtered out PCR duplicates using Picard tools (http://picard.sourceforge.net). We then applied the GATK pipeline (McKenna et al., 2010) to identify high-confidence positions with bi-allelic expression on the X chromosome (chrX) and on chromosome 7 (chr7) by base quality score recalibration, indel realignment, and SNP

discovery across each individual sample using standard hard filtering parameters to minimize the number of false positives (QUALITY≥100, coverage ≥10, SNP within the dbSNP database (build 137), AF=0.5). We normalized the number of X-linked bi-allelic positions by the number of positions potentially interrogated by our pipeline, *i.e* the number of known SNPs (dbsnp_137.b37) on the chrX with a coverage≥10 reads. We used the number of bi-allelic positions on chr7 as quality metrics of the RNA-seq samples (as an autosome should be bi-allelically expressed in all samples) and kept for further analysis cells with a number of bi-allelic positions on chr7 $n_{chr7}>19$. We represented the normalized number of X-linked bi-allelic positions using boxplots and pooling the samples according to stage (day for dataset #2 and developmental stage dor dataset #1) or to *XACT* expression (negative for log2 < -9 and positive for log2 ≥ -9). We compared the distribution of these numbers between categories using Wilcoxon's rank test. We also compared the numbers of X-linked bi-allelic positions to the numbers of positions found for chr1, chr 2 and chr 7.

We also repeated the analysis performed by Petropoulos *et al.* to compare with our results (Petropoulos et al., 2016). Briefly, we used SAMtools mpileup (Li et al., 2009) to retrieve allelic read counts for positions in dbSNP (build 137) with a minimal coverage of 3 and a threshold for the allelic ratio between the major and minor allele of 10 to separate mono and bi-allelic expressed positions. We computed a 'bi-allelic' ratio between the chrX and chr7, which is a ratio of the fractions of positions with bi-allelic calls over the total number of positions with sufficient coverage for each chromosome (Supplementary Fig. 1g). With a threshold for the allelic ratio of 10, we do not find any statistical difference in these ratios between different stages as in Petropoulos *et al.*. However when we decreased this threshold to 3 (the minor allele with at least 25% of the reads), we find a significant decrease of the number of these positions on the chrX compared to autosome along stage progression. The differences between observations made with an allelic ratio of 10 or 3 indicate that there is an imbalance between the two X chromosomes starting at E6, as there are less positions with equivalent transcription from each X.

RNA-FISH on human embryos

French Biomedecine Agency authorization was obtained for the experimental use of supernumerary cryopreserved embryos resulting from infertility treatment and donated for research (RE 10-032R/RE 12-012R). Written consents were obtained from the couples that their cryopreserved embryos could be used for the research. Briefly, after removal of the *zona pellucida*, embryos were rinsed in embryo culture medium and transferred onto a Denhardt’s solution-coated coverslip and air-dried for 30 min at RT. Embryos were then fixed in 3% paraformaldehyde for 10 min at RT and permeabilised on ice in PBS with 0.5% Triton X-100 and 2mM Vanadyl Ribonucleoside Complex (New England Biolabs) for 8 to 12 min depending on the embryo stage, and then progressively dehydrated in ethanol. The coverslips were kept in 70% ethanol at -20°C before RNA-FISH. SpectrumGreen or SpectrumRed-dUTP (Vysis) were used to generate by nick translation labeled probes for *XIST* (a 10kb fragment corresponding to *XIST* exon 1, (gift from Dr. Carolyn Brown, Department of Medical Genetics, University of British Columbia, Vancouver, British Columbia, Canada) and for *XACT* (RP11-35D3, BACPAC). Prior to hybridization, 0.1µg of probe was ethanol-precipitated together with 10 µg of salmon sperm (Thermo Fisher Scientific) for *XIST* and 10µg of human Cot-1 (Thermo Fisher Scientific) for *XACT*, washed twice in 70% ethanol and resuspended in formamide (Sigma-Aldrich). The probes were denaturated at 75°C for 7 min, mixed with an equal quantity of 2x hybridization buffer (4XSSC, 20% Dextran Sulfate, 2mg/ml BSA, 2mM Vanadyl Ribonucleoside Complex (New England Biolabs))

and kept on ice before use. Embryos were hybridized with labeled probes overnight at 37°C in a dark and humid chamber. After three washes in 50% formamide/2x SSC, three washes in 2x SSC at 42°C, coverslips were counterstained with DAPI (1ug/ml), mounted and viewed under the fluorescence microscope. A 200M Axiovert (Zeiss) fluorescence microscope equipped with an ApoTome system was used for image acquisition and the generation of optical sections in 3D. Sequential z-axis images were collected in 0.3μm steps. At the blastocyst stage, when possible, we were able to distinguish cells corresponding to the trophectoderm or the inner cell mass according to their morphological aspect.

Cell culture of primed and naïve human embryonic stem cells

WA09/H9 NK2 and WA01/H1 primed hESC and their naïve counterparts were kindly provided by Austin Smith with permission from WiCell (Takashima et al., 2014). Primed WIBR3 hESC were kindly provided by Rudolph Jaenisch (Theunissen et al., 2014). Primed hESC were cultured on CF1 irradiated mouse embryonic fibroblasts (MEF) in Advanced DMEM containing 20% Knockout Serum Replacement (Thermo Fisher Scientific) supplemented with 2mM L-Glutamine (Thermo Fisher Scientific), 0.1mM β-mercaptoethanol (Sigma-Aldrich), 1x Penicillin/Streptomycin (Thermo Fisher Scientific), 1x Non-Essential Amino Acids (Thermo Fisher Scientific) and 4ng/ml FGF2 (WT-MRC Cambridge Stem Cell Institute). Naïve H9 and H1 hESC were cultured on CF1 MEF in a 1:1 mixture of DMEM/F12 and Neurobasal (Thermo Fisher Scientific), 0.5x N2-supplement (Thermo Fisher Scientific), 0.5x B27-supplement (Thermo Fisher Scientific), 1x Non-Essential Amino Acids (Thermo Fisher Scientific), 2mM L-Glutamine (Thermo Fisher Scientific), 1x Penicillin/Streptomycin (Thermo Fisher Scientific), 0.1mM β-mercaptoethanol (Sigma-Aldrich), 1μM PD0325901, 1μM CHIR99021, 20ng/ml human LIF (all from WT-MRC Cambridge Stem Cell Institute) and 2μM Gö6983 (Sigma-Aldrich). Naïve WIBR3 hESC were converted from primed hESC as follows. Primed hESC were dissociated into single cells with Accutase and seeded at a density of 2x104 cells/cm2 in primed hESC medium containing 10μM Y-27632 on MEF-coated plates. The following day, media was changed to 5iLA, which consists of a 1:1 mixture of DMEM/F12 and Neurobasal (Thermo Fisher Scientific), 1x N2-supplement (Thermo Fisher Scientific), 1x B27-supplement (Thermo Fisher Scientific), 1x Non-Essential Amino Acids (Thermo Fisher Scientific), 2mM L-Glutamine (Thermo Fisher Scientific), 1x Penicillin/Streptomycin (Thermo Fisher Scientific), 0.1mM β-mercaptoethanol (Sigma-Aldrich), 50μg/ml Bovine Serum Albumin (Thermo Fisher Scientific), 1μM PD0325901 (WT-MRC Cambridge Stem Cell Institute), 0.5μM IM-12 (Cell Guidance Systems), 0.5μM SB590885 (Cell Guidance Systems), 1μM WH4-023 (Cell Guidance Systems), 10μM Y-27632 (Cell Guidance Systems), 20ng/ml human LIF (WT-MRC Cambridge Stem Cell Institute) and 10ng/ml Activin A (WT-MRC Cambridge Stem Cell Institute). Cells were passaged after six days with Accutase at a 1:4 split ratio and expanded for several passages in 5iLA media on MEF-coated plates. All cells were cultured in 5% O2, 5% CO2 at 37°C. Authentication of hESC was achieved by confirmation of expression of pluripotency gene and protein markers. Cells were routinely verified as mycoplasma-free using a PCR-based assay. No cell lines used in this study were found in the database of commonly misidentified cell lines that is maintained by ICLAC and NCBI Biosample.

Transitioning primed cells using RSeT

To transition primed cells, we started from human-embryo derived H9 (P40-P52) and WIBR2 (P55-P67) cells, which were maintained in conventional feeder-free PSC culture conditions with MTeSR1™

(STEMCELL technologies) on Matrigel hES-qualified Matrix (Corning) coated plates. For resetting of hESCs into a naïve-like state, cells were cultured in RSeT™ medium, following the guidelines of the manufacturer (STEMCELL technologies). Briefly, hESCs in mTeSR1™ were split with Gentle Dissociation solution (STEMCELL technology) and cell aggregates were plated onto a layer of MEF feeders with MTeSR1™ under normoxic conditions (20% O2, 5%CO2). The following day, the medium was replaced with RSeT™ and cells were further cultivated under hypoxic conditions (5% O2, 5% CO2). Medium was changed daily. Cells were split every 4-6 days, after dissociation with TrypLE express (Thermo Fisher Scientific). After each passage, cells were incubated on RSeT™ in the presence of Rho-associated kinase inhibitor (ROCKi) (Y-27632, EMD Millipore), until the following medium change. Transcriptional changes indicative of pluripotency (Grow et al., 2015; Weinberger et al., 2016) where assessed by qRT-PCR.

Allelic expression analysis of X-linked transcripts in primed and naïve H9 cells

We used published RNA-seq datasets for primed and naïve H9 cells: GSE60945 (Takashima et al., 2014). We took advantage of the clonal XCI pattern of H9 cells (Mitjavila-Garcia et al., 2010; Shen et al., 2008) to analyze the data in an allelic manner. We had previously identified a set of SNPs on the X chromosome (198 SNPs on the X chromosome, corresponding to 78 genes) that could be used for allelic analysis (Vallot et al., 2015). We considered a transcript as bi-allelic when at least 25% of reads were originating from the minor allele (allelic ratio 3:1). We computed the allelic information by gene and counted the number of bi-allelic and mono-allelic expressed gene in both primed and naïve H9 cells and compared them using a Fisher's exact test.

RNA and DNA-FISH on cell lines

Cells were fixed at room temperature in 3% Paraformaldehyde (Electron Microscopy Science)/PBS for 10min and permeabilized as previously described (Vallot et al., 2015) in CSK buffer supplemented with 0.5% Triton (Sigma-Aldrich), 2mM EGTA (Sigma-Aldrich) and 2mM VRC (New England Biolabs) for 5min, and washed 3 times in ice-cold 70% Ethanol. SpectrumGreen or SpectrumRed-labeled probes (Vysis) were generated by nick translation for human *XIST* (see above), mouse *Xist* (p510), *XACT* (RP11-35D3, BACPAC), *ATRX* (RP11-42M11, BACPAC Resource), *FGD1* (RP11-625D4, BACPAC) and *POLA1* (RP11-1104L9, BACPAC). For RNA and DNA-FISH, all probes generated from BACs were precipitated with human Cot-1 DNA (Thermo Fisher Scientific) and the *XIST*-probe with Salmon Sperm DNA (Thermo Fisher Scientific), resuspended in formamide and denatured for 7min at 75°C. Probes are then diluted in an equal volume of 2X Hybridization Buffer (4XSSC, 20% Dextran Sulfate, 2mg/ml BSA, 2mM Vanadyl Ribonucleoside Complex). Cot-1 precipitated probes are additionally pre-incubated 15min at 37°C.

For RNA-FISH, coverslips were dehydrated in 90% and 100% ethanol and incubated overnight with probe at 37°C. After three 50% formaldehyde/2XSSC washes and three 2XSSC washes at 42°C for 4min, coverslips were mounted in Vectashield plus DAPI (Vector Laboratories).

For DNA-FISH, coverslips were dehydrated in 70%, 90% and 100% ethanol and adapted in 2XSCC at 80°C. Coverslips were denaturated for 10min at 80°C in 70% formamide/2XSSC (pH=7.2) and were dehydrated in 70%, 90% and 100% ethanol and incubated overnight with probe at 37°C. After three 2XSSC washes at 45°C and three 0.1XSSC washes at 60°C for 4min, coverslips were mounted in Vectashield plus DAPI.

Microscopy and image analysis

All images were taken on a fluorescence DMI-6000 inverted microscope with a motorized stage (Leica) and with a CCD Camera HQ2 (Roper Scientifics) controlled by the Metamorph 7.04 software (Roper Scientifics) using a HCX PL APO 100X oil objective (numerical aperture, 1.4, Leica). Optical Z-sections were collected at 0.5µm steps through each nucleus at different wavelengths depending on the probes used (DAPI [360nm, 470nm], FITC [470nm, 525nm], cy3 [550nm, 570nm], Texas Red [596nm, 612nm] and cy5 [647nm, 668nm]); approximately 30 optical sections per nucleus were collected. Stacks were processed using ImageJ 1.46 (Abramoff et al., 2004), and throughout the manuscript the 3D-FISH experiments are represented as a 2D-projection of the stacks (maximum projection).

We designed in-house ImageJ macros to extract quantitative information from 3D-images and perform subsequent image analysis in R. We automatically extracted from each nucleus the fluorescence value of each pixel from *XACT* and *XIST* signal, as well as Yen's threshold for each signal. In R, we imported the matrices of fluorescence values and their associated thresholds, and limited our analysis to pixels above respective thresholds for *XACT* and *XIST* images. We first converted the positions of pixels in µm, according to the camera and objective settings. We computed the dispersion of *XIST* RNA, by comparing the cumulative volume of the signal to the theoretical spherical volume it could occupy based on the maximal radial distance (Fig. 3G). We computed the cumulative volume occupied by *XACT* RNA, by adding the volume of all pixels with intensity above threshold. To compare the localization of *XACT* and *XIST* RNAs, for each image (corresponding to an individual X chromosome), we compared the intensity of fluorescence on the union of pixels with signal above threshold for *XACT* or *XIST* signal through the calculus of a Spearman's correlation score and p-value for at least n=90 images.

Immunofluorescence and RNA-FISH

Naïve cells were cultured on 12mm coverslips and were first fixed in 3% Paraformaldehyde/PBS for 10min at RT and then permeabilized for 5min at RT with CSK buffer (NaCl, MgCl2, Sucrose, PIPES, pH=6.8) supplemented with 0.5% Triton and 2mM EGTA. Cells were incubated 45min with 0.2% Gelatin/PBS, 45min with rabbit polyclonal primary antibody (anti-H3K4me2 (ab7766 Abcam), anti-H3K27me3 (07-449 Millipore), anti-H3K9me3 (gift from from Pr. Prim Singh (Cowell et al., 2002) and anti-H3K27Ac (ab4729 Abcam)) diluted in 0.2% Gelatin/PBS, washed 3 times in PBS, incubated 30min with an Alexa Fluor 488nm anti-rabbit secondary antibody (Life Technologies) and washed 3 times with PBS. Cells were fixed in 3% Paraformaldehyde/PBS for 10min at RT before proceeding to RNA-FISH as described above. Immunofluorescence and RNA-FISH signals were simultaneously observed using a confocal microscope DMI6000 TCS SP5 (Leica). To evaluate the colocalization between *XACT*, *XIST* and histone marks, we compared grey values along line scans within single z-sections.

Analysis of ChIP-seq datasets

We used our previously published H3K27me3 and H3K9me3 ChIP-seq datasets for primed *XIST*+ hESCs (GSE62562) as well as published H3K27me3 and H3K9me3 ChIP-seq datasets for naïve *XIST*+ hESCs (GSE59435 and GSE84382). We analysed fastq files as previously described (Vallot et al., 2015). We subdivided the X chromosome in 100kbp windows, and computed for each window the number of reads from the ChIP and the input experiment. For the scatterplots, we defined the enrichment as

the ratio of the number of reads in the ChIP over the input in these 100kbp-windows. We compared ratios between cell lines using Spearman's correlation scores.

Generation of mouse ES cells with *XACT* or *FGD1* human genes

Female LF2 ES cell lines were grown in Dulbecco's modified Eagle medium (DMEM, Thermo Fisher Scientific), 15% Fetal calf serum (FCS, Thermo Fisher Scientific) and 1000 U/mL LIF (Millipore), on gelatin-coated dishes and in absence of feeder cells. Cell passaging (1:6 split) was done by enzymatic treatment with trypsin (Trypsin-EDTA 0.05%, Thermo Fisher Scientific) for 4 minutes. Cells were transfected using Lipofectamine2000 (Thermo Fisher Scientific). For random BAC integration, cells were transfected with a BAC encompassing part of the *XACT* locus including its promoter (RP11-110P23, chrX: 113085338- 113248350) and a plasmid encoding a Neomycin resistance gene (ratio 1:3). For targeted integration, LF2 cells were co-transfected with a *XACT* BAC (or *FGD1* BAC RP11-625D4) together with a plasmid (pSpCas9(BB)-2A-GFP (PX458), gift from Feng Zhang (Addgene plasmid # 48138)) containing the Cas9 gene, and a gRNA. We used two gRNAs, designed with CRIPSR Design (http://crispr.mit.edu), targeting the mouse region syntenic to the human *XACT* locus; the first one targeting chrX: 146088618-146088637, and the second one targeting chrX: 146096270-146096289. Twelve hours post-transfection, cells were plated in 10-cm dishes and left under 0.25µg/ml G418 selection (Thermo Fisher Scientific) for 10 days. Clones were screened for *XACT* or FGD1 insertion by PCR and DNA-FISH, and for *XACT* or *FGD1* expression by RNA-FISH. We determined the copy number of the BAC in each clone, using quantitative real-time PCR. We diluted pure BAC DNA within LF2 genomic DNA to achieve artificial preparations with various ratios of the BAC copy number versus the genomic DNA (100 copies to 1 copy). We normalized the BAC copy number to the copy number of the *Chic1* gene to account for variations in the measurement of the DNA concentrations of the samples.

Differentiation of mouse ES clones

Differentiation experiments were carried out on gelatin-coated dishes at a density of 10 000 cells per cm2, in Dulbecco modified Eagle medium (DMEM, Thermo Fisher Scientific) and 10% Fetal calf serum (FCS, Thermo Fisher Scientific). RNAs were extracted at day 0, 2, 4 and 7 of differentiation from biological triplicates (independent differentiation experiments). Cells were fixed at day 0, 2, 4 and 7 for RNA-FISH.

Knockdown using LNA Gapmers

We designed three different Locked Nucleic Acid (LNA) Gapmers targeting *XACT* and a control scramble LNA Gapmer devoid of target RNA using the Exiqon online tool (Exiqon). LNAs were delivered at 50nM via lipofection using the transfection reagent RNAimax (Invitrogen), according to manufacturer recommendations. We kept for further analysis of the T9_XACT clone, the only LNA Gapmer ('LNA_XACT') and the scrambled Gapmer ('LNA_SCR') that triggered an efficient knockdown of the XACT transgene at day 2 after transfection. We transfected the T9_XACT clone two days prior to the initiation of differentiation (d-2), the day of the differentiation (d0) and two days after the initiation of differentiation (d2). We collected cells for RNA and RNA-DNA FISH experiments at day -2, day 0, day 2 and day 4, for three independent experiments.

Metaphase spreads and DNA-FISH on mouse clones

Metaphase spreads were prepared as described (Naim et al., 2013). Spreads were then fixed in 0.25% PFA for 10min at RT, dehydrated and denatured 5min at 75°C in 70% Formamide/2X SSC. Mouse X chromosome paint (Metasystems) was denatured 2min at 75°C and applied on slides.

RNA extraction and RT-qPCR

Total RNA was extracted from all cells using trizol (Thermo Fisher Scientific). RNA was treated with Dnase I (Roche) to remove DNA contamination. One µg of total RNA was used for reverse transcription, using the SuperScript II kit (Thermo Fisher Scientific). mRNA expression levels were evaluated using real-time quantitative PCR (RT-qPCR) with the SYBR Green kit on an ABI PRISM 7500 real-time thermal cycler (Applied Biosystems). All samples were run in duplicate. RNA expression levels for the transcripts of interest were normalized against the reference gene *Arpo* for mouse samples and to *U6* for human samples, according to the $2^{-\Delta Ct}$ method.

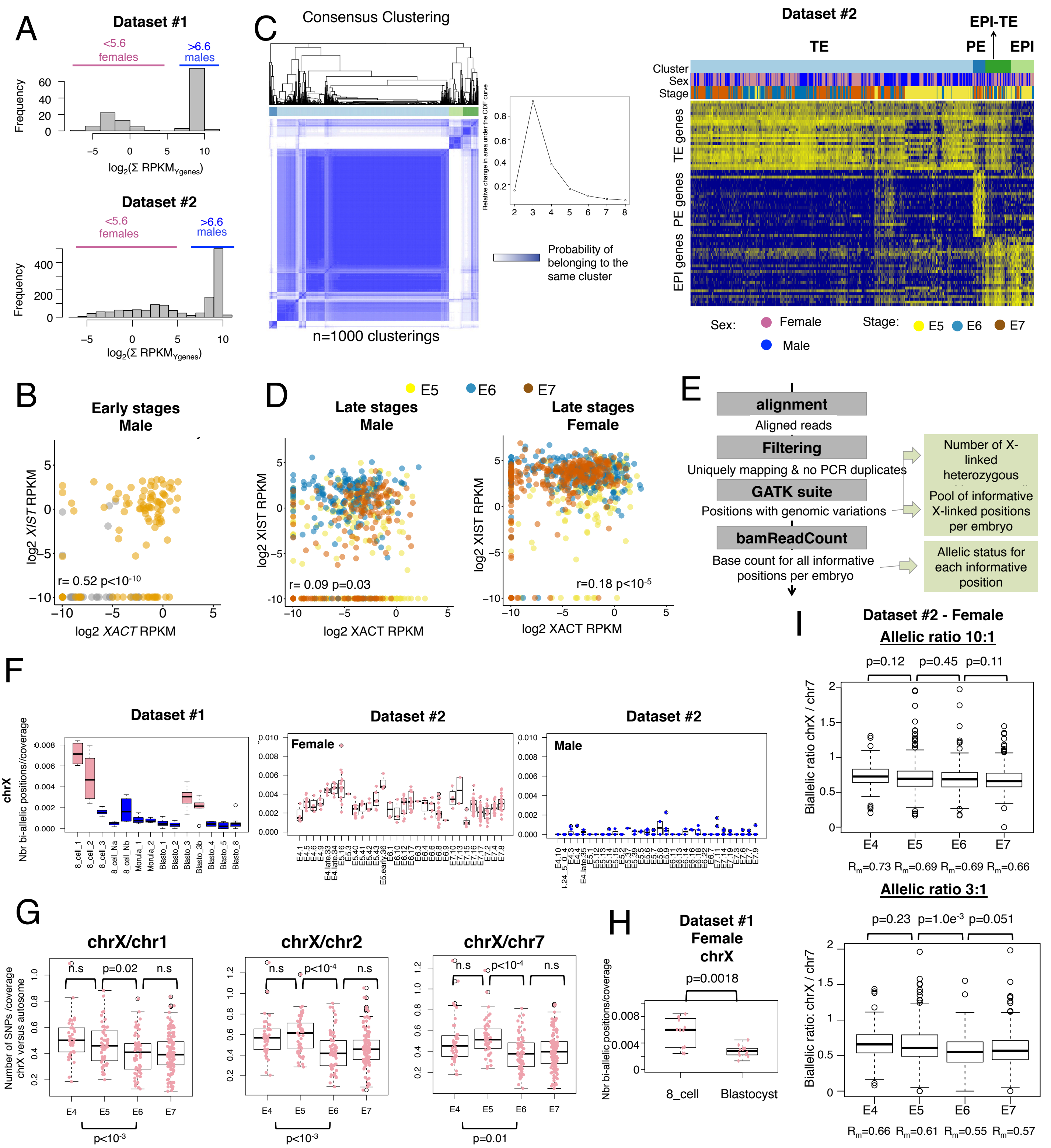

Figure S1, related to Figure 1: **Linking *XACT* and *XIST* expression levels to sex, lineage commitment and X chromosome inactivation in the early steps of human development**.

(**A**) Determination of the sex of each embryo for datasets #1 and 2 as in Petropoulos *et al.* [2]; the histograms display the bimodal distribution of the sum of the RPKM of Y-linked genes for each cell. Cells with $\log2(\Sigma RPKM_{Ygenes})>6.6$ were assigned as males, whereas cells with $\log2(\Sigma RPKM_{Ygenes})<5.6$ were assigned as females. (**B**) Plot of *XACT* versus *XIST* expression levels (log2 RPKM) in early stages male cells of dataset #2 (E3, E4 and early E5), with corresponding Spearman's correlation score and p-value. (**C**) Consensus clustering approach to assess lineage commitment within cells from E5, E6 and E7 embryos. Left panel: Consensus clustering (for k=4 clusters) of all cells (n=1000 hierarchical clustering were performed on 80% randomly chosen samples and 80% randomly chosen genes to evaluate the stability of the clustering) and plot of the area under the CDF curve for each choice of number of cluster (k=2 to 8). The relative change in area does not reach a plateau while k is increasing, indicating that the signature does not permit a fully stable partition of the samples. We chose to partition the samples in k=4 clusters, considering that the corresponding consensus clustering displays a stable partition for PE and part of the EPI and TE samples, moreover k>4 does not resolve the proper partition of samples with a mixed signature. Right panel: Heatmap illustrating the expression pattern of the 75-gene signature (25 genes characterizing each lineage, TE, EPI and PE) and the repartition of the late stage samples into 4 clusters. Each cluster was named according to the expression pattern of the genes characteristic of each lineage. (**D**) Plots of *XACT* versus *XIST* expression levels (log2 RPKM) in late stages male cells of dataset #2 (E5, E6 and E7), with corresponding Spearman's correlation score and p-value. The color code illustrates the stage in the left panel, and the commitment lineage in the right panels (see (c)). (**E**) Schematic summary of the pipeline used to probe X chromosome inactivation in both datasets. Grey boxes relate to tools whereas green boxes relate to results. (**F**) The boxplots represent for each dataset the normalized number of X-linked bi-allelic positions detected per cell grouped per embryo. Each points within the boxplots corresponds to a single cell of the given embryo (pink for females and blue for males). (**G**) Same analysis as in Fig.1E, the numbers of X-linked bi-allelic positions are compared to the numbers for different autosomes (chr1, chr2 and chr7), n.s stands for '*not significant*' ($p>0.05$). (**H**) Same as in Fig.1E for female samples of dataset #1. (**I**) Computation of the number of bi-allelic positions using a different pipeline (Petropoulos *et al.* 2016), based on the counting across known SNPs on the X-chromosome; boxplots represent the distribution of chr X to chr 7 ratio of normalized number of bi-allelic positions across days of development for an allelic ratio of 10:1 (upper panel) as in Petropoulos et al. 2016 and an allelic ratio of 3:1 (lower panel). Comparisons of distributions within stages were quantified using Wilcoxon's [illegible]

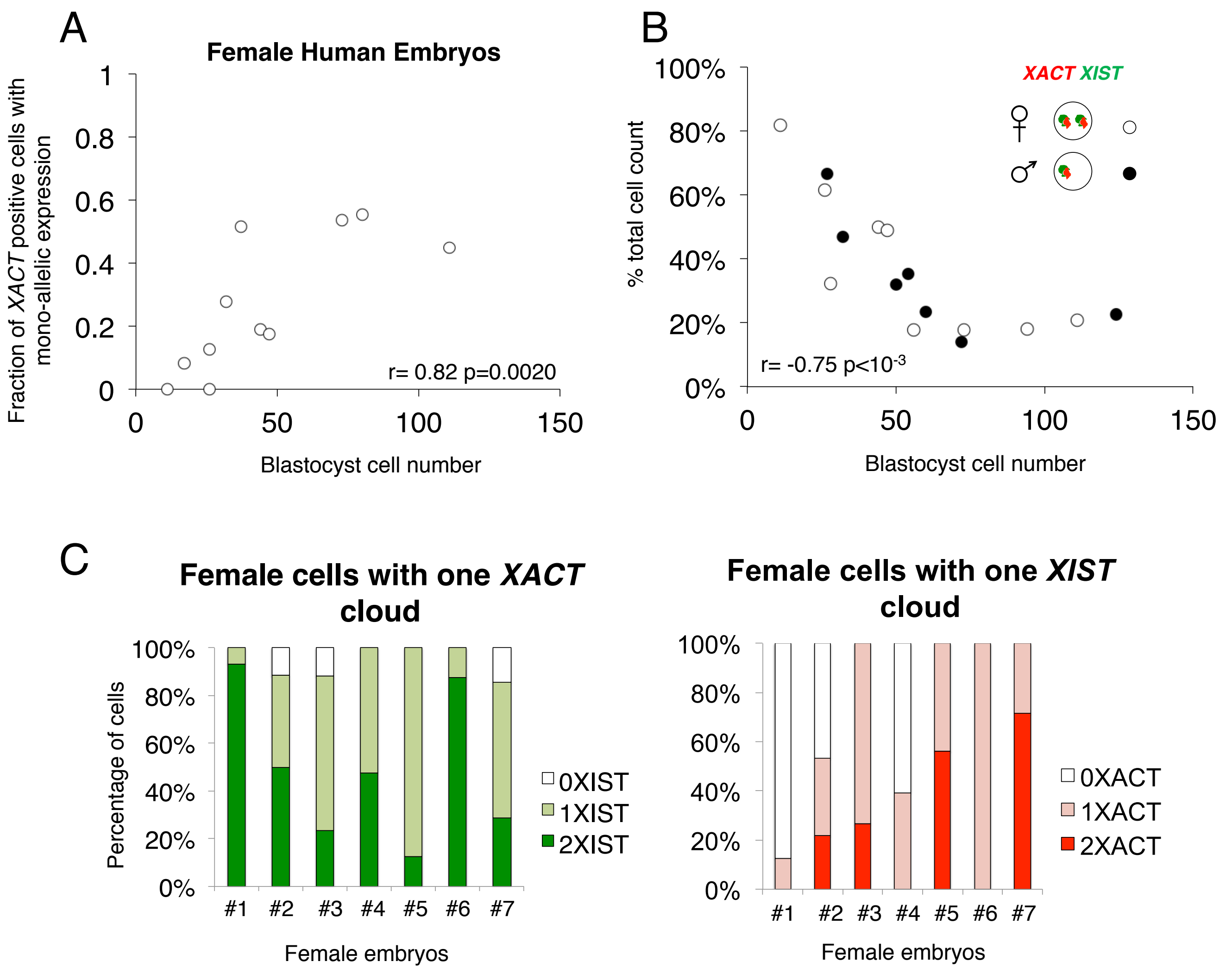

Figure S2, related to Figure 2: ***XIST* and *XACT* expression pattern in human blastocysts.**
(A) The plot represents the fraction of *XACT* positive female cells showing mono-allelic accumulation, according to the blastocyst cell number. **(B)** Plot of the percentage of cells accumulating *XIST* and *XACT* RNAs versus the blastocyst cell number per male (dark circles) and female (white circles) embryos. The correlation between measures was assessed in both cases using a Spearman's correlation test. (**C**) Histograms representing for each female embryo the expression pattern of *XIST* in cells with mono-allelic expression of *XACT*. Same representation for the expression pattern of *XACT* in cells with mono-allelic expression of *XIST*.

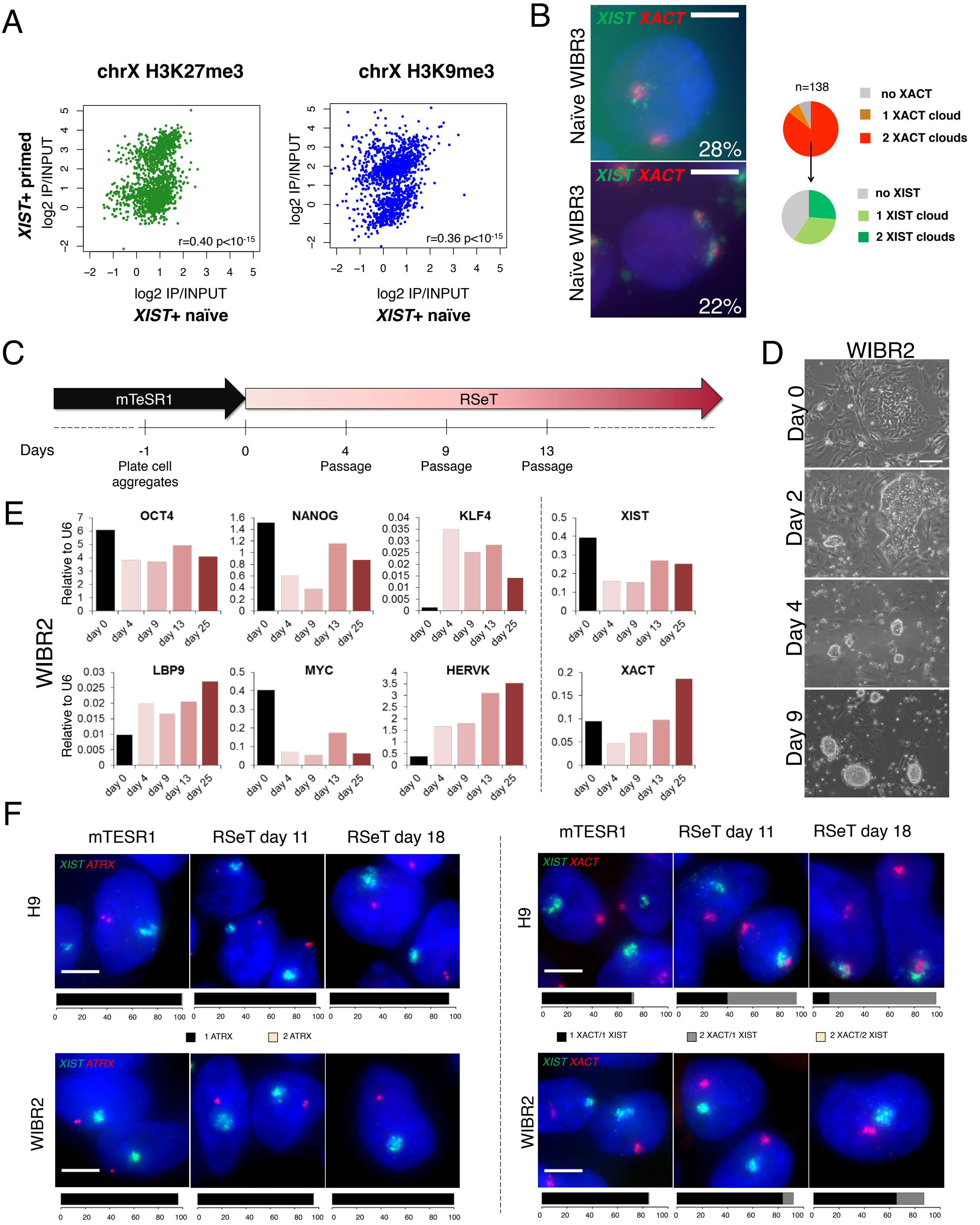

Figure S3, related to Figure 3: **Additional characterization of reprogrammed hESCs. (A)** Scatter plot comparing H3K27me3 and H3K9me3 log2 enrichment ratios on the X chromosome between *XIST*-expressing primed hESCs (H9 (Vallot et al., 2015)) and naive hESCs (WIBR2 for H3K27me3 (Theunissen et al., 2014) and WIBR3 for H3K9me3 (Theunissen et al., 2016)). Log2 ratios were compared using Pearson correlation tests and the associated p-value was calculated using random permutations of the data sets. **(B)** RNA-FISH analysis revealing *XACT* (red) and *XIST* (green) expression pattern in naïve female WIBR3. (**C**) Schematic view of the procedure to transition hESCs from mTeSR to RSeT, H9 and WIBR2. (**D**) Representative bright field images of WIBR2 cells at days 0, 2, 4 and 9 of the transition. (**E**) Left panel: qRT-PCR quantification of markers of naïve and primed pluripotency during the transition of WIBR2 from mTeSR to RSeT. The histograms display the enrichment compared to U6. Right panel: qRT-PCR analysis for *XIST* and *XACT*. (**F**) Left panel: RNA-FISH analysis with *ATRX* (red) and *XIST* (green) probes. Displayed pictures correspond to the most frequent expression pattern at each day of RSeT transition. The barplots beneath the pictures represent the percentage of cells with each expression pattern. Right panel: RNA-FISH analysis with *XACT* (red) and *XIST* (green) probes. The white scale bar corresponds to 5 μm.

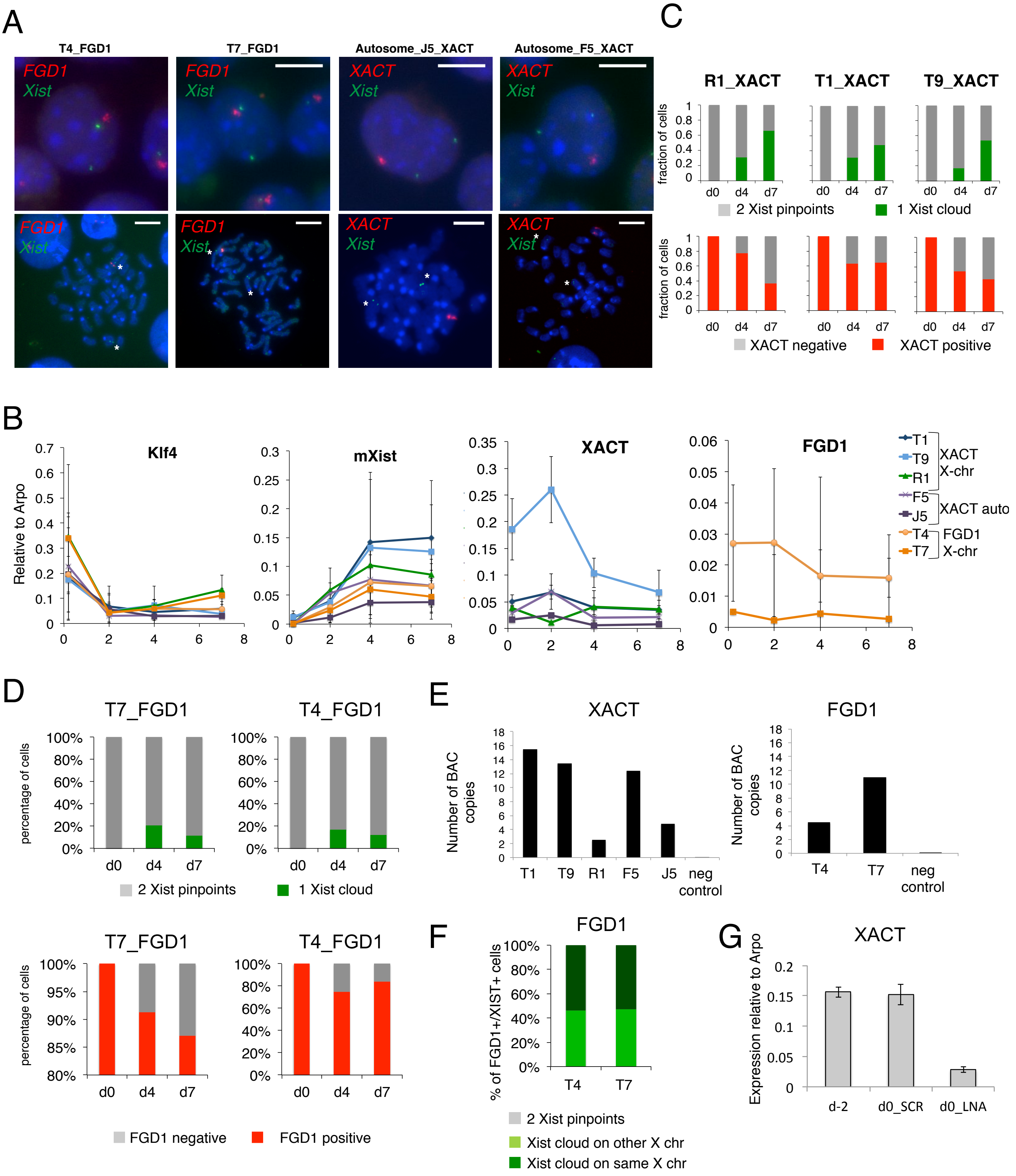

Figure S4, related to Figure 4: **Insertion of human *XACT* or *FGD1* transgenes in mESCs**.
(**A**) Assessment by RNA-FISH of *Xist* (in green) and transgene *(FGD1* or *XACT,* in red*)* expression in interphase nuclei of undifferentiated mouse ES cells (upper panels) and of their localization on metaphase chromosomes (lower panels) in clones with targeted (T4 and T7) integration of *FGD1* and autosomal integration of *XACT* (F5 and J5). The white scale bars correspond to 5µm for interphase nuclei and to 10 µm for metaphases. (**B**) qRT-PCR analysis of *Klf4*, *Xist*, *FGD1* and *XACT* expression in differentiating R1, T1, T9, T4, T7, J5 and F5 clones. (**C**) Quantification of *Xist* and *XACT* RNA-FISH patterns in R1, T1 and T9 *XACT*-transgenic clones. (**D**) Quantification of *Xist* and *FGD1* RNA-FISH patterns in T7 and T4 *FGD1*-transgenic clones. (**E**) The barplot represents the number of BAC copies per cells for *XACT* (left panel) and *FGD1* (right panel), as assessed by qPCR using as a reference serial dilutions of the BAC DNA within LF2 ES cells DNA, and adjusting for differences in DNA concentration with *Chic1* copy number. (**F**) Assessment of the chromosome of origin for *Xist* expression related to *FGD1* integration. In T4 and T7 clones, *Xist* is equally up-regulated from the WT or the transgenic X. A Fisher's exact test was used to compare numbers of nuclei with *Xist* accumulation on WT or transgenic X to a 50/50 repartition. (**G**) Quantification of *XACT* expression using RT-qPCR for 3 independent KD experiments using control LNA Gapmer (SCR) and the LNA Gapmer targeting XACT (LNA) in the T9 clone. Expression is calculated relatively to *Arpo*.

Supplementary references

Abramoff, M.D., Magalhaes, P.J., and Ram, S.J. (2004). Image Processing with ImageJ. Biophotonics International *11*, 36-42.

Anders, S., Pyl, P.T., and Huber, W. (2015). HTSeq--a Python framework to work with high-throughput sequencing data. Bioinformatics *31*, 166-169.

Blakeley, P., Fogarty, N.M., del Valle, I., Wamaitha, S.E., Hu, T.X., Elder, K., Snell, P., Christie, L., Robson, P., and Niakan, K.K. (2015). Defining the three cell lineages of the human blastocyst by single-cell RNA-seq. Development *142*, 3151-3165.

Cowell, I.G., Aucott, R., Mahadevaiah, S.K., Burgoyne, P.S., Huskisson, N., Bongiorni, S., Prantera, G., Fanti, L., Pimpinelli, S., Wu, R.*, et al.* (2002). Heterochromatin, HP1 and methylation at lysine 9 of histone H3 in animals. Chromosoma *111*, 22-36.

Grow, E.J., Flynn, R.A., Chavez, S.L., Bayless, N.L., Wossidlo, M., Wesche, D.J., Martin, L., Ware, C.B., Blish, C.A., Chang, H.Y.*, et al.* (2015). Intrinsic retroviral reactivation in human preimplantation embryos and pluripotent cells. Nature *522*, 221-225.

Kim, D., Pertea, G., Trapnell, C., Pimentel, H., Kelley, R., and Salzberg, S.L. (2013). TopHat2: accurate alignment of transcriptomes in the presence of insertions, deletions and gene fusions. Genome Biol *14*, R36.

Li, H., Handsaker, B., Wysoker, A., Fennell, T., Ruan, J., Homer, N., Marth, G., Abecasis, G., and Durbin, R. (2009). The Sequence Alignment/Map format and SAMtools. Bioinformatics *25*, 2078-2079.

McKenna, A., Hanna, M., Banks, E., Sivachenko, A., Cibulskis, K., Kernytsky, A., Garimella, K., Altshuler, D., Gabriel, S., Daly, M.*, et al.* (2010). The Genome Analysis Toolkit: a MapReduce framework for analyzing next-generation DNA sequencing data. Genome Res *20*, 1297-1303.

Mitjavila-Garcia, M.T., Bonnet, M.L., Yates, F., Haddad, R., Oudrhiri, N., Feraud, O., Magniez, A., Makhlouf, M., Vallot, C., Rougeulle, C.*, et al.* (2010). Partial reversal of the methylation pattern of the X-linked gene HUMARA during hematopoietic differentiation of human embryonic stem cells. J Mol Cell Biol *2*, 291-298.

Petropoulos, S., Edsgard, D., Reinius, B., Deng, Q., Panula, S.P., Codeluppi, S., Plaza Reyes, A., Linnarsson, S., Sandberg, R., and Lanner, F. (2016). Single-Cell RNA-Seq Reveals Lineage and X Chromosome Dynamics in Human Preimplantation Embryos. Cell *165*, 1012-1026.

Shen, Y., Matsuno, Y., Fouse, S.D., Rao, N., Root, S., Xu, R., Pellegrini, M., Riggs, A.D., and Fan, G. (2008). X-inactivation in female human embryonic stem cells is in a nonrandom pattern and prone to epigenetic alterations. Proc Natl Acad Sci U S A *105*, 4709-4714.

Takashima, Y., Guo, G., Loos, R., Nichols, J., Ficz, G., Krueger, F., Oxley, D., Santos, F., Clarke, J., Mansfield, W.*, et al.* (2014). Resetting transcription factor control circuitry toward ground-state pluripotency in human. Cell *158*, 1254-1269.

Theunissen, T.W., Powell, B.E., Wang, H., Mitalipova, M., Faddah, D.A., Reddy, J., Fan, Z.P., Maetzel, D., Ganz, K., Shi, L.*, et al.* (2014). Systematic identification of culture conditions for induction and maintenance of naive human pluripotency. Cell Stem Cell *15*, 471-487.

Vallot, C., Ouimette, J.F., Makhlouf, M., Féraud, O., Pontis, J., Côme, J., Martinat, C., Bennaceur-Griscelli, A., Lalande, M., and Rougeulle, C. (2015). Erosion of X Chromosome Inactivation in Human Pluripotent Cells Initiates with XACT Coating and Depends on a Specific Heterochromatin Landscape. Cell Stem Cell *16*, 533-546.

Weinberger, L., Ayyash, M., Novershtern, N., and Hanna, J.H. (2016). Dynamic stem cell states: naive to primed pluripotency in rodents and humans. Nat Rev Mol Cell Biol *17*, 155-169.

Wilkerson, M.D., and Hayes, D.N. (2010). ConsensusClusterPlus: a class discovery tool with confidence assessments and item tracking. Bioinformatics *26*, 1572-1573.

Xue, Z., Huang, K., Cai, C., Cai, L., Jiang, C.Y., Feng, Y., Liu, Z., Zeng, Q., Cheng, L., Sun, Y.E.*, et al.* (2013). Genetic programs in human and mouse early embryos revealed by single-cell RNA sequencing. Nature *500*, 593-597.

Yan, L., Yang, M., Guo, H., Yang, L., Wu, J., Li, R., Liu, P., Lian, Y., Zheng, X., Yan, J., *et al.* (2013). Single-cell RNA-Seq profiling of human preimplantation embryos and embryonic stem cells. Nature structural & molecular biology *20*, 1131-1139.
